# Efficient Synthesis of a New Family of 2,6-Disulfanyl-9-selenabicyclo[3.3.1]nonanes

**DOI:** 10.3390/molecules26102849

**Published:** 2021-05-11

**Authors:** Maxim V. Musalov, Vladimir A. Potapov, Svetlana V. Amosova

**Affiliations:** A. E. Favorsky Irkutsk Institute of Chemistry, Siberian Division of The Russian Academy of Sciences, 1 Favorsky Str., 664033 Irkutsk, Russia; musalov_maxim@irioch.irk.ru (M.V.M.); amosova@irioch.irk.ru (S.V.A.)

**Keywords:** 2,6-disulfanyl-9-selenabicyclo[3.3.1]nonanes, transannular addition, 9-selenabicyclo[3.3.1]nonane-2,6-dithiolate anion, isothiouronium salts, 2,6-dibromo-9-selenabicyclo[3.3.1]nonane, selenium dibromide

## Abstract

The efficient synthesis of a new family of 2,6-disulfanyl-9-selenabicyclo[3.3.1]nonanes in high yields has been developed based on 9-selenabicyclo[3.3.1]nonane-2,6-dithiolate anion generated from bis-isothiouronium salt of 2,6-dibromo-9-selenabicyclo[3.3.1]nonane. The derivatives of 2,6-disulfanyl-9-selenabicyclo[3.3.1]nonane containing alkyl, allyl and benzyl moieties have been prepared in 90–99% yields by nucleophilic substitution of 9-selenabicyclo[3.3.1]nonane-2,6-dithiolate anion with alkyl, allyl and benzyl halides. The reaction of nucleophilic addition of 9-selenabicyclo[3.3.1]nonane-2,6-dithiolate anion to alkyl propiolates afforded 2,6-di(vinylsulfanyl)-9-selenabicyclo[3.3.1]nonanes. The conditions for regio- and stereoselective addition of 9-selenabicyclo[3.3.1]nonane-2,6-dithiolate anion to a triple bond of alkyl propiolates have been found. To date, not a single representative of 2,6-disulfanyl-9-selenabicyclo[3.3.1]nonanes has been described in the literature.

## 1. Introduction

The importance of chemistry of heterocyclic compounds for the development of organic medicinal and pharmaceutical chemistry is difficult to overestimate. A lion’s share of modern drugs contains heterocyclic moieties in their structures [1,2]. The discovery of many novel drugs is closely related to the development of chemistry of heterocyclic compounds. Heterocyclic derivatives exhibit various types of biological activity [1,2]. Many distinguished scientists have made important contributions to modern chemistry of heterocyclic compounds [1,2,3,4].

Selenium is a micronutrient for mammals and an essential trace element nutrient for humans that functions as cofactor for glutathione peroxidase and certain forms of thioredoxin reductase [5,6,7]. Organoselenium heterocycles display a variety of biological activities, including antibacterial, antifungal, antitumor, anti-inflammatory, neuroprotective and glutathione peroxidase-like actions [8,9,10,11,12,13,14,15].

Selenium heterocyclic compound Ebselen shows anti-inflammatory, neuroprotective and glutathione peroxidase-like activities [13,14,15]. This compound finds application as an anti-inflammatory agent. Ebselen is also used for the treatment and prevention of cardiovascular diseases and ischemic stroke.

The anchimeric assistance effect of selenium in comparison with the effect of sulfur and nitrogen atoms has been quantitatively estimated using 2,6-dichloro-9-selenabicyclo [3.3.1]nonane, 2,6-dichloro-9-thia- and 2,6-dichloro-9-azabicyclo[3.3.1]nonane as model substrates [16]. Based on the determination of the absolute and relative rates of nucleophilic substitution of chlorine in these compounds, it has been established that the anchimeric assistance effect of the selenium atom is more than two orders of magnitude greater than the effect of the sulfur and nitrogen atoms. 2,6-Dichloro-9-selenabicyclo[3.3.1]nonane has been obtained by the transannular addition of selenium dichloride to *cis*,*cis*-1,5-cyclooctadiene [16].

The biochemical potential of 9-selenabicyclo[3.3.1]nonanes has not yet been revealed; however, it is known that its sulfur and nitrogen analogues exhibit a variety of biological activities [17,18,19,20,21,22,23,24,25,26,27].

Antimicrobial coatings containing the 9-thiabicyclo[3.3.1]nonane moiety have been developed [17]. The resulting surfaces displayed antibacterial and antifungal activities.

**Figure 1:**
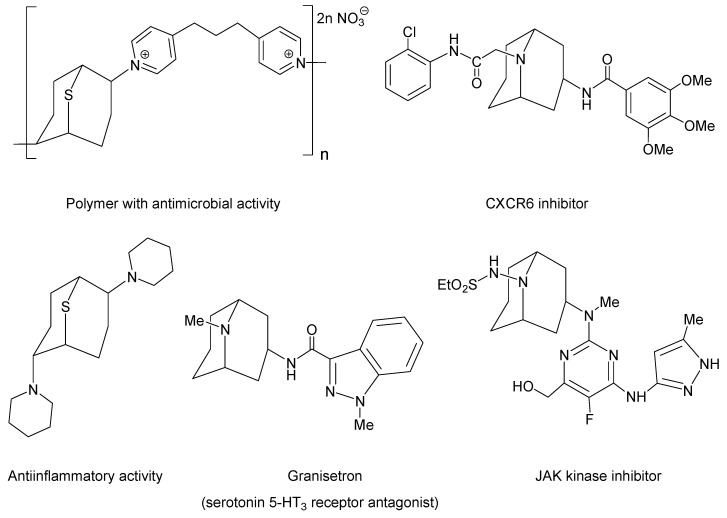
Known biologically active derivatives of 9-thiabicyclo[3.3.1]nonane [18,19] and 9-azabicyclo[3.3.1]nonane [20,26,27].

The 9-thiabicyclo[3.3.1]nonane derivatives, which were obtained by nucleophilic substitution of halogen in 2,6-dichloro-9-thiabicyclo[3.3.1]nonane, displayed anti-inflammatory activity [18] (Figure 1). Polycation polymers containing the 9-thiabicyclo[3.3.1]nonane unit showed antimicrobial activity [19] (Figure 1). These polymers inhibited the growth of bacteria at low concentration (e.g., the minimum inhibitory concentration in PBS buffer is 0.12–0.5 μg/mL against *Bacillus subtilis* and *Escherichia coli*.

The medicine granisetron containing the 9-azabicyclo[3.3.1]nonane moiety is a serotonin 5-HT_3_ receptor antagonist [20] (Figure 1). This drug is used for preventing postoperative nausea and vomiting.

Novel polycationic materials based on 9-thia-, 9-aza-, and 9-selena[3.3.1]bicyclononanes have been synthesized and proposed as DNA-transfecting polymers [21,22,23]. An important desirable feature of DNA-transfecting polymers is the ability to degrade into non-toxic components after cellular uptake of a DNA-polymer complex. Cationic polymers composed of repeating units of 9-thia-, 9-aza-, and 9-selena[3.3.1]bicyclononanes have been found to show high transfection efficacy in a galactosidase assay.

Polymers and resins containing the 9-thiabicyclo[3.3.1]nonane and 9-selenabicyclo[3.3.1]nonane units have been applied for preparation of materials with high refractive index [24,25].

Derivatives of 9-azabicyclo[3.3.1]nonane have been proposed as CXCR6 receptor inhibitors [26] and JAK kinase inhibitors [27] (Figure 1).

The JAK kinase inhibitors are relatively new drugs exhibiting significant therapeutic advances. JAK kinase inhibitors are a type of medication that functions by inhibiting the activity of one or more of the Janus kinase family of enzymes. The JAK kinase inhibitors may have therapeutic application in the treatment of cancer, inflammatory diseases and various autoimmune diseases [27].

Selenium dichloride and dibromide were first involved in the synthesis of organoselenium compounds in 2003 [28,29]. The reaction of selenium dihalides with dimethyldiethynylsilane led to 3,6-dihalo-4,4-dimethyl-1,4-selenasilafulvenes [28]. Currently organic synthesis based on selenium dihalides is an intensively developing area of research [30,31,32,33,34,35,36,37,38,39]. Annulation reactions of selenium dihalides with unsaturated arenes gave various condensed heterocyclic compounds [40,41,42,43,44]. The addition of selenium dihalides to alkenes and alkynes afforded bis(2-haloalkyl) selenides [45,46] and bis(2-halovinyl) selenides in high yields [47,48,49,50,51,52]. Novel heterocycles [53,54,55,56,57,58,59,60,61,62] have been obtained by reactions of selenium dihalides with divinyl chalcogenides [63,64,65].

**Scheme 1:**
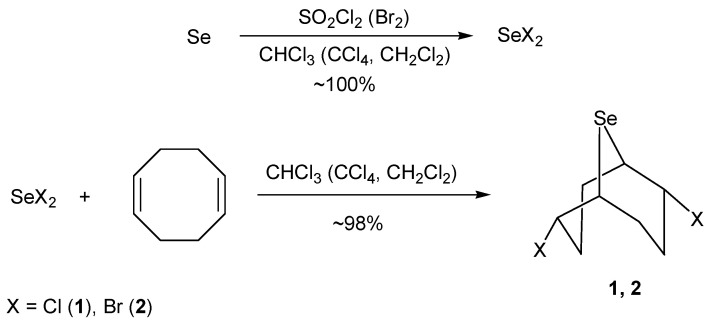
Transannular addition of selenium dihalides to *cis*,*cis*-1,5-cyclooctadiene affording 2,6-dihalo-9-selenabicyclo[3.3.1]nonanes (**1**, **2**).

Extending our studies of the reactions of selenium dihalides with linear dienic compounds [53,54,55,56,57,58,59,60,61,62,66], we explored their addition to cyclodienes. The reaction of selenium dihalides with *cis*,*cis*-1,5-cyclooctadiene occurred as transannular addition affording 2,6-dihalo-9-selenabicyclo[3.3.1]nonanes in almost quantitative yields (Scheme 1) [16,67,68].

We studied nucleophilic substitution reaction of bromine in compound **2** by pyridine [69,70]. Dipiridinium salt, 2,6-dipyridiumyl-9-selenabicyclo[3.3.1]nonane dibromide, was obtained in near quantitative yield [69,70]. The biological activity of this compound as a medicine for metabolic correction in the vaccination process and its effect on immunogenesis were studied [69]. It was found that this compound considerably diminished the pathological effect caused by the action of the tularemia vaccine in experimental animals and significantly reduced the reactogenicity of the brucellosis vaccine. Based on these results, 2,6-dipyridiumyl-9-selenabicyclo[3.3.1]nonane dibromide was proposed as a promising drug for metabolic correction in the vaccination process [69].

## 2. Results and Discussion

Nucleophilic substitution reactions of bromine in compound **2** by sulfur-centered nucleophiles have not been studied and not a single representative of 2,6-disulfanyl-9-selenabicyclo[3.3.1]nonanes has been described in the literature.

**Figure 2:**
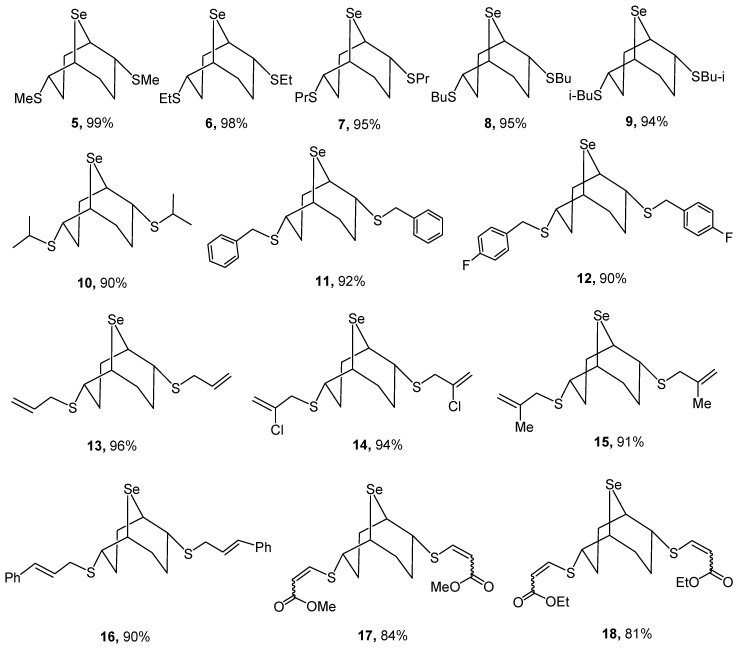
A new family of compounds, 2,6-disulfanyl-9-selenabicyclo[3.3.1]nonane derivatives.

The efficient synthesis of a new family of 2,6-diorganylsulfanyl-9-selenabicyclo[3.3.1]nonanes has been developed in the present work (Figure 2). Theoretically, these compounds can be obtained by nucleophilic substitution reactions of bromine in compound **2** by organylthiols. However, we found a more efficient approach to 2,6-diorganylsulfanyl-9-selenabicyclo[3.3.1]nonanes, which includes the preparation of bis-isothiouronium salt from compound **2** and thiourea. This approach opens up more synthetic possibilities and allows obtaining not only nucleophilic substitution products but also products of nucleophilic addition to a triple bond.

**Scheme 2:**
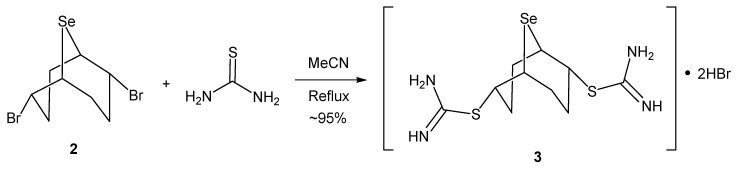
Synthesis of bis-isothiouronium salt **3** by the reaction of compound **2** with thiourea in acetonitrile.

Bis-isothiouronium salt **3** was prepared in 95% yield by the reaction of thiourea with compound **2** in acetonitrile under reflux. Bis-isothiouronium salt **3** precipitated under the reaction conditions and can be easily isolated (Scheme 2).

**Scheme 3:**
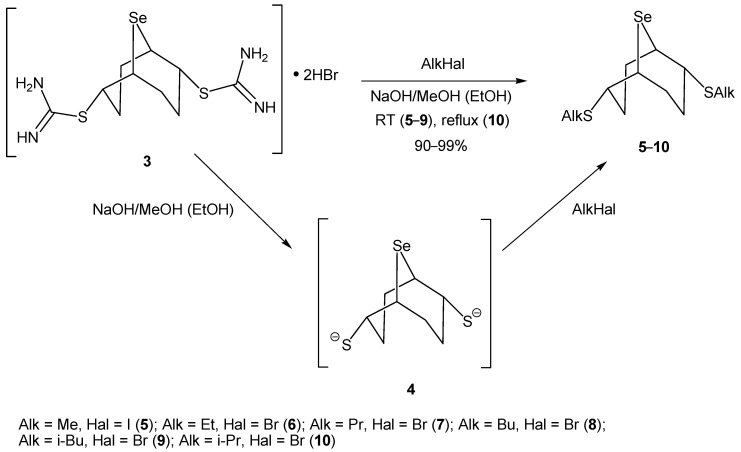
Synthesis of 2,6-di(alkylsulfanyl)-9-selenabicyclo[3.3.1]nonanes (**5**–**10**)) by nucleophilic substitution of alkyl halides by 9-selenabicyclo[3.3.1]nonane-2,6-dithiolate anion generated from bis-isothiouronium salt **3**.

The action of alkalis on bis-isothiouronium salt **3** led to generation of 9-selenabicyclo[3.3.1]nonane-2,6-dithiolate anion **4**, which was involved in nucleophilic substitution reactions with a variety of alkylating reagents (Scheme 3). The conditions for efficient synthesis of 2,6-dialkylsulfanyl-9-selenabicyclo[3.3.1]nonanes have been found. In a typical procedure, sodium hydroxide was added to a methanol or ethanol solution containing alkylating reagent (MeI, EtBr, PrBr, BuBr, i-BuBr).

The reaction proceeded under mild condition at room temperature in such “green solvents” as methanol or ethanol affording the target product **5**–**9** in 94–99% yields without additional purification (Scheme 3).

In the case of the reaction of dithiolate anion **4** with isopropyl bromide at room temperature, the corresponding product **10** was formed only in 52% yield. However, carrying out the process under reflux made it possible to accelerate this reaction and to obtain isopropyl derivative **10** in 90% yield after purification on a short column with silica gel (Scheme 3).

**Scheme 4:**
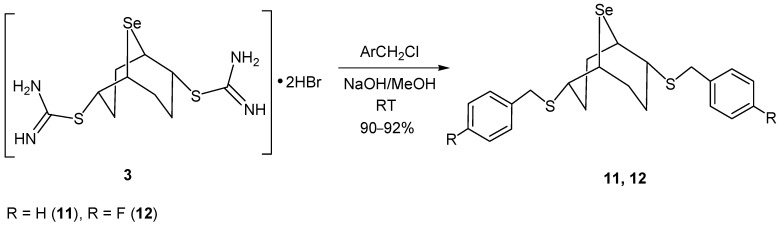
Synthesis of 2,6-di(benzylsulfanyl)-9-selenabicyclo[3.3.1]nonanes (**11**, **12**) from bis-isothiouronium salt **3** and benzyl halides.

Although chlorine is usually displaced more slowly than bromine in nucleophilic substitution, the reactions of bis-isothiouronium salt **3** with benzyl and 4-fluorobenzyl chlorides proceeded smoothly at room temperature leading to 2,6-di(benzylsulfanyl)-9-selenabicyclo[3.3.1]nonanes **11**, **12** in 90–92% yields (Scheme 4). It is worth noting that introduction of fluorine to organic molecules is usually favorable from the viewpoint of possible manifestation of biological activity and a number of modern important drugs contain the fluorine atom [71].

**Scheme 5:**
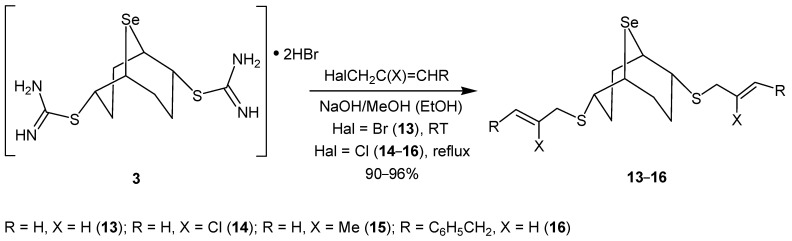
Synthesis of 2,6-di(allylsulfanyl)-9-selenabicyclo[3.3.1]nonanes (**13**–**16**) from bis-isothiouronium salt **3** and allyl halides (allyl bromide, 2,3-dichloro-1-propene, 3-chloro-2-methyl-1-propene, *E*-3-chloro-1-propenylbenzene).

Allyl bromide easily reacted with bis-isothiouronium salt **3** at room temperature, leading to 2,6-di(allylsulfanyl)-9-selenabicyclo[3.3.1]nonane **13** in 96% yields (Scheme 4). However, in the case of the reactions of bis-isothiouronium salt **3** with substituted allyl chlorides (3-chloro-2-methyl-1-propene, 2,3-dichloro-1-propene, *E*-3-chloro-1-propenylbenzene) under the same conditions at room temperature, corresponding products were obtained in 60–72% yields. In order to increase the yields of the products, the reactions of bis-isothiouronium salt **3** with substituted allyl chlorides were carried out with heating (50–60 °C). This made it possible to accelerate the reaction and to obtain compounds **14**–**16**, which were isolated in 90–94% yields by purification on a short column with silica gel (Scheme 5).

Finally, we realized the addition of 9-selenabicyclo[3.3.1]nonane-2,6-dithiolate anion to activated triple bond of alkyl propiolates. The conditions for efficient regio- and stereoselective reaction of bis-isothiouronium salt **3** with alkyl propiolates were established.

We found that it is advisable to carry out the reaction of bis-isothiouronium salt **3** with methyl propiolate in methanol and the process with ethyl propiolate advantageously to conduct in ethanol. Otherwise, the formation of some by-products derived from the interconversion of methyl and ethyl esters (the transesterification reaction in the presence of bases). Besides, the amount of alkali should be reduced by 2 times in comparison with the previous conditions for nucleophilic substitution reactions.

**Scheme 6:**
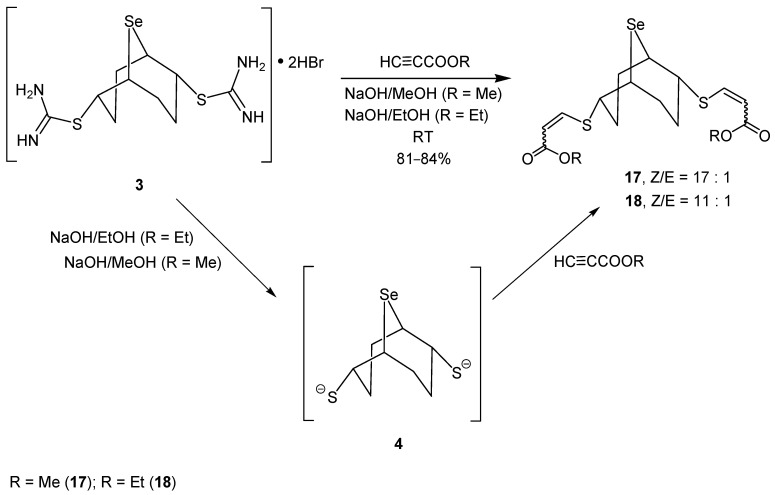
Synthesis of 2,6-di(vinylsulfanyl)-9-selenabicyclo[3.3.1]nonanes (**17**, **18**) by nucleophilic addition of 9-selenabicyclo[3.3.1]nonane-2,6-dithiolate anion to alkyl propiolates.

Thus, the reaction of nucleophilic addition of 9-selenabicyclo[3.3.1]nonane-2,6-dithiolate anion **4** to methyl and ethyl propiolates proceeded in a regio- and stereoselective manner, affording 2,6-di(vinylsulfanyl)-9-selenabicyclo[3.3.1]nonanes **17** (a ratio of Z/E isomers ~17:1) in 84% yield and **18** (a ratio of Z/E isomers ~11:1) in 81% yield (Scheme 6).

The obtained products represent a new family of compounds, 2,6-disulfanyl-9-selenabicyclo[3.3.1]nonane derivatives (Figure 2), with promising biological activity.

The structural assignments of the synthesized compounds were made using ^1^H and ^13^C-NMR spectroscopy and confirmed by elemental analysis. The signals of the CHSe group in ^13^C NMR spectra of compounds **5**–**18** manifested themselves in the region 29.2–30.5 ppm (^1^*J*_C-Se_ = 51.5–54.3 Hz). Stereoconfiguration of the vinyl group in compounds **17** and **18** was assigned based on the values of proton spin–spin coupling constants (^3^*J*_H-H_), which are 10.0–10.2 Hz for (*Z*)-isomers and 15.2–15.3 Hz for (*E*)-isomers.

## 3. Experimental Section

### 3.1. General Information

The ^1^H (400.1 MHz) and ^13^C (100.6 MHz) NMR spectra (see Supplementary Materials) were recorded on a Bruker DPX-400 spectrometer (Bruker BioSpin GmbH, Rheinstetten, Germany) in CDCl_3_ (compounds **5**–**18**) and *d*_6_-DMSO (compounds **3**) solutions and referred to the residual solvent peaks of CDCl_3_ (δ = 7.27 and 77.16 ppm in ^1^H- and ^13^C-NMR, respectively) or *d*_6_-DMSO (δ = 2.50 and 39.5 ppm in ^1^H- and ^13^C-NMR, respectively). Elemental analysis was performed on a Thermo Scientific Flash 2000 Elemental Analyzer (Thermo Fisher Scientific Inc., Milan, Italy). Melting points were determined on a Kofler Hot-Stage Microscope PolyTherm A apparatus (Wagner & Munz GmbH, Munich, Germany). The organic solvents were dried and distilled according to standard procedures. Silica gel (Alfa Aesar, 0.06–0.20 mm (70–230 mesh) was used for column chromatography.

### 3.2. Synthesis of Bis-Isothiouronium Salt **3**

*2,6-Bis[amino(iminio)methylsulfanyl]-9-selenabicyclo[3.3.1]nonane dibromide* (**3**). Thiourea (1.52 g, 2 mmol) was added to a solution of compound **2** (3 g, 0.865 mmol) in acetonitrile (120 mL). The mixture was stirred at room temperature for 2 h and then heated under reflux with stirring for 5 h. The formation of white precipitate was observed. Precipitated product was filtered, washed with cold hexane and dried in vacuum, giving bis-isothiouronium salt **3** (4.10 g, 95% yield) as a white powder; mp 219–220 °C.

^1^H-NMR (400 MHz, *d*_6_-DMSO): 2.03–2.17 (m, 4H, CH_2_CHS, CH_2_CHSe), 2.32–2.41 (m, 2H, CH_2_CHS), 2.55–2.62 (m, 2H, CH_2_CHSe), 3.15–3.19 (m, 2H, CHS), 4.70–4.76 (m, 2H, CHSe), 9.02–9.30 (m, 8H, CN_2_H_4_). ^13^C-NMR (100 MHz, *d*_6_-DMSO): 27.9 (CH_2_CHSe), 28.5 (CH_2_CHS), 29.1 (CHSe), 49.3 (CHS), 167.7 (C=N).

Anal. calcd for C_10_H_20_ N_4_S_2_Br_2_Se (499.19): C 24.06, H 4.04, N 11.22, S 12.85, Br 32.01, Se 15.82%. Found: C 23.91, H 3.99, N 11.20, S 12.80, Br 32.43, Se 15.98%.

### 3.3. Synthesis of Compounds **5**–**18**

*2,6-Bis(methylsulfanyl)-9-selenabicyclo[3.3.1]nonane* (**5**). A solution of methyl iodide (0.26 g, 1.8 mmol) in ethanol (1 mL) was added to a solution of bis-isothiouronium salt **3** (0.35 g, 0.7 mmol) in ethanol (4 mL). Then a solution of sodium hydroxide (80%, 0.2 g, 4 mmol) in ethanol (3 mL) was added dropwise to the reaction mixture. The mixture was stirred for 8 h at room temperature. Methylene chloride (15 mL) and cold water (15 mL) were added to the reaction mixture. The mixture was transferred to a separatory funnel and the organic layer was separated. The mixture was additionally extracted with methylene chloride (2 × 10 mL), the organic phase was dried over CaCl_2_ and the solvent was removed by a rotary evaporator. The residue was dried in vacuum, giving product **5** (0.195 g, 99% yield) as a white powder; mp 64–65 °C.

^1^H NMR (400 MHz, CDCl_3_): 1.73–1.84 (m, 2H, CH_2_CHS), 1.93–2.02 (m, 2H, CH_2_CHSe), 2.03 (s, 6H, CH_3_), 2.12–2.22 (m, 2H, CH_2_CHS), 2.64–2.73 (m, 2H, CH_2_CHSe), 2.97–3.02 (m, 2H, CHS), 3.47–3.54 (m, 2H, CHSe). ^13^C NMR (100 MHz, CDCl_3_): 14.25 (CH_3_), 28.8 (CH_2_CHSe), 29.2 (CHSe, ^1^*J*_Se-C_ = 51.5 Hz), 29.4 (CH_2_CHS), 48.3 (CHS).

Anal. calcd for C_10_H_18_S_2_Se (281.34): C 42.69, H 6.45, S 22.79, Se 28.07%. Found: C 42.91, H 6.46, S 22.98, Se 28.47%.

*2,6-Bis(ethylsulfanyl)-9-selenabicyclo[3.3.1]nonane* (**6**). A solution of ethyl bromide (0.28 g, 2.6 mmol) in methanol (1 mL) was added to a solution of bis-isothiouronium salt **3** (0.43 g, 0.86 mmol) in methanol (5 mL). Then, a solution of sodium hydroxide (80%, 0.25 g, 5 mmol) in methanol (4 mL) was added dropwise to the reaction mixture. The mixture was stirred overnight (14 h) at room temperature. Methylene chloride (20 mL) and cold water (20 mL) were added to the reaction mixture. The mixture was transferred to a separatory funnel and the organic layer was separated. The mixture was additionally extracted with methylene chloride (2 × 10 mL), the organic phase was dried over CaCl_2_ and the solvent was removed by a rotary evaporator. The residue was dried in vacuum, giving product **6** (0.26 g, 98% yield) as a white powder; mp 59–60 °C.

^1^H NMR (400 MHz, CDCl_3_): 1.15 (t, 6H, CH_3_, ^3^*J*_H-H_ = 7.4 Hz), 1.76–1.88 (m, 2H, CH_2_CHS), 1.91–1.98 (m, 2H, CH_2_CHSe), 2.12–2.20 (m, 2H, CH_2_CHS), 2.41–2.53 (m, 4H, SCH_2_CH_3_), 2.64–2.73 (m, 2H, CH_2_CHSe), 2.93–2.97 (m, 2H, CHS), 3.56–3.62 (m, 2H, CHSe). ^13^C NMR (100 MHz, CDCl_3_): 14.9 (CH_3_), 24.8 (CH_2_CH_3_), 29.0 (CH_2_CHSe), 29.9 (CH_2_CHS), 30.0 (CHSe, ^1^*J*_Se-C_ = 52.7 Hz), 48.3 (CHS).

Anal. calcd for C_12_H_22_S_2_Se (309.39): C 46.58, H 7.17, S 20.73, Se 25.52%. Found: C 46.31, H 7.15, S 20.88, Se 25.86%.

*2,6-Bis(propylsulfanyl)-9-selenabicyclo[3.3.1]nonane* (**7**) was obtained as a colourless viscous oil (0.276 g, 95% yield) from bis-isothiouronium salt **3** (0.43 g, 0.86 mmol), propyl bromide (0.32 g, 2.6 mmol) and sodium hydroxide (80%, 0.25 g, 5 mmol) in methanol under the same conditions as compound **6.**

^1^H NMR (400 MHz, CDCl_3_): 0.89 (t, 6H, CH_3_, ^3^*J*_H-H_ = 7.4 Hz), 1.43–1.55 (m, 4H, CH_2_CH_3_), 1.76–1.88 (m, 2H, CH_2_CHSe), 1.91–1.99 (m, 2H, CH_2_CHS), 2.09–2.22 (m, 2H, CH_2_CHSe), 2.36–2.49 (m, 4H, CH_2_S), 2.65–2.74 (m, 2H, CH_2_CHS), 2.92–2.97 (m, 2H, CHS), 3.54–3.58 (m, 2H, CHSe). ^13^C NMR (100 MHz, CDCl_3_): 13.4 (CH_3_), 23.1 (CH_2_CH_3_), 29.0 (CH_2_CHSe), 30.0 (CH_2_CHS), 30.1 (CHSe, ^1^*J*_Se-C_ = 52.6 Hz), 33.0 (CH_2_S), 48.8 (CHS).

Anal. calcd for C_14_H_26_S_2_Se (337.45): C 49.83, H 7.77, S 19.00, Se 23.40%. Found: C 49.65, H 7.63, S 19.34, Se 23.86%.

*2,6-Bis(butylsulfanyl)-9-selenabicyclo[3.3.1]nonane* (**8**) was obtained as a colourless viscous oil (0.299 g, 95% yield) from bis-isothiouronium salt **3** (0.43 g, 0.86 mmol), butyl bromide (0.35 g, 2.6 mmol) and sodium hydroxide (80%, 0.25 g, 5 mmol) in methanol under the same conditions as compound **6.**

^1^H NMR (400 MHz, CDCl_3_): 0.82 (t, 6H, CH_3_, ^3^*J*_H-H_ = 7.2 Hz), 1.27–1.36 (m, 4H, CH_2_CH_3_), 1.43–1.50 (m, 4H, CH_2_CH_2_S), 1.74–1.88 (m, 2H, CH_2_CHSe), 1.92–1.99 (m, 2H, CH_2_CHS), 2.12–2.22 (m, 2H, CH_2_CHSe), 2.40–2.52 (m, 4H, CH_2_S), 2.66–2.74 (m, 2H, CH_2_CHS), 2.94–2.99 (m, 2H, CHS), 3.54–3.59 (m, 2H, CHSe). ^13^C NMR (100 MHz, CDCl_3_): 13.7 (CH_3_), 22.0 (CH_2_CH_3_), 29.1 (CH_2_CHSe), 30.1 (CH_2_CHS), 30.1 (CHSe, ^1^*J*_Se-C_ = 52.6 Hz), 30.7 (CH_2_CH_2_S), 32.0 (CH_2_S), 48.9 (CHS).

Anal. calcd for C_16_H_30_S_2_Se (365.50): C 52.58, H 8.27, S 17.55, Se 21.60%. Found: C 52.75, H 8.19, S 17.74, Se 21.42%.

*2,6-Bis(isobutylsulfanyl)-9-selenabicyclo[3.3.1]nonane* (**9**) was obtained as a colourless viscous oil (0.296 g, 94% yield) from bis-isothiouronium salt **3** (0.43 g, 0.86 mmol), isobutyl bromide (0.35 g, 2.6 mmol) and sodium hydroxide (80%, 0.25 g, 5 mmol) in methanol under the same conditions as compound **6.**

^1^H NMR (400 MHz, CDCl_3_): 0.98 (d, 12H, CH_3_, ^3^*J*_H-H_ = 6.7 Hz), 1.70–1.81 (m, 2H, CH_2_CHS), 1.86–1.97 (m, 2H, CH_2_CHSe), 2.02–2.10 (m, 2H, CH_2_CHS), 2.20–2.30 (m, 2H, CH_2_CHSe), 2.36–2.48 (m, 4H SCH_2_), 2.76–2.82 (m, 2H, CHCH_2_S), 3.02–3.05 (m, 2H, CHS), 3.59–3.65 (m, 2H, CHSe). ^13^C NMR (100 MHz, CDCl_3_): 22.1 (CH_3_), 22.3 (CH_3_), 29.0 (CHCH_3_), 29.3 (CH_2_CHSe), 30.2 (CH_2_CHS), 30.3 (CHSe, ^1^*J*_Se-C_ = 52.1 Hz), 40.4 (CH_2_S), 48.3 (CHS).

Anal. calcd for C_16_H_30_S_2_Se (365.50): C 52.58, H 8.27, S 17.55, Se 21.60%. Found: C 52.34, H 8.15, S 17.41, Se 21.84%.

*2,6-Bis(isopropylsulfanyl)-9-selenabicyclo[3.3.1]nonane* (**10**). A solution of isopropyl bromide (0.32 g, 2.6 mmol) in methanol (1 mL) was added to a solution of compound **3** (0.43 g, 0.86 mmol) in methanol (5 mL). Then a solution of sodium hydroxide (80%, 0.25 g, 5 mmol) in methanol (4 mL) was added dropwise and the mixture was refluxed for 3 h. Methylene chloride (20 mL) and cold water (20 mL) were added to the reaction mixture. The mixture was transferred to a separatory funnel and the organic layer was separated. The mixture was additionally extracted with methylene chloride (2 × 10 mL), the organic phase was dried over CaCl_2_ and the solvent was removed by a rotary evaporator. The residue was subjected to column chromatography on silica gel (eluent: hexane, then hexane/chloroform 1:10). Compound **10** (0.262 g, 90% yield) was isolated as a colourless viscous oil.

^1^H NMR (400 MHz, CDCl_3_): 1.19 (d, 12H, CH_3_, ^3^*J*_H-H_ = 6.7 Hz), 1.78–1.91 (m, 2H, CH_2_CHS), 1.98–2.18 (m, 4H, CH_2_CHSe, CH_2_CHS), 2.41–2.53 (m, 4H, SCH_2_CH_3_), 2.65–2.74 (m, 2H, CH_2_CHSe), 2.78–2.99 (m, 4H, CHS, CH_3_CHS), 3.56–3.62 (m, 2H, CHSe).

^13^C NMR (100 MHz, CDCl_3_): 23.6, 23.9 (CH_3_), 29.3 (CH_2_CHSe), 30.3 (CH_2_CHS), 30.5 (CHSe, ^1^*J*_Se-C_ = 53.6 Hz), 33.9 (CH_3_CHS), 47.4 (CHS).

*2,6-Bis(benzylsulfanyl)-9-selenabicyclo[3.3.1]nonane* (**11**) was obtained from bis-isothiouronium salt **3** (0.43 g, 0.86 mmol), benzyl bromide (0.41 g, 2.4 mmol) and sodium hydroxide (80%, 0.25 g, 5 mmol) in methanol under the same conditions as compound **6**. The product was purified by column chromatography on silica gel (eluent: hexane, then hexane/chloroform 1:10). Compound **11** (0.342 g, 92% yield) was isolated as a white powder; mp 71–72 °C.

^1^H NMR (400 MHz, CDCl_3_): 1.81–1.97 (m, 4H, CH_2_CHS, CH_2_CHSe), 2.09–2.18 (m, 2H, CH_2_CHS), 2.67–2.74 (m, 2H, CH_2_CHSe), 2.88–2.94 (m, 2H, CHS), 3.50–3.56 (m, 2H, CHSe), 3.65–3.72 (m, 4H, SCH_2_Ar), 7.15–7.23 (m, 2H, CH_Ar_), 7.21–7.32 (m, 8H, CH_Ar_). ^13^C NMR (100 MHz, CDCl_3_): 29.2 (CH_2_CHSe), 29.7 (CH_2_CHS), 29.8 (CHSe, ^1^*J*_Se-C_ = 52.9 Hz), 35.6 (ArCH_2_S), 48.4 (CHS), 127.1 (C_Ar_), 128.6 (CH_Ar_), 128.7 (CH_Ar_), 138.4 (C_Ar_).

Anal. calcd for C_22_H_26_S_2_Se (433.53): C 60.95, H 6.04, S 14.79, Se 18.21%. Found: C 60.56, H 6.01, S 14.96, Se 18.54%.

*2,6-Bis(4-fluorobenzylsulfanyl)-9-selenabicyclo[3.3.1]nonane* (**12**) was obtained from bis-isothiouronium salt **3** (0.43 g, 0.86 mmol), 4-fluorobenzyl bromide (0.456 g, 2.4 mmol) and sodium hydroxide (80%, 0.25 g, 5 mmol) in methanol under the same conditions as compound **6**. The product was purified by column chromatography on silica gel (eluent: hexane, then hexane/chloroform 1:10). Compound **12** (0.363 g, 90% yield) was isolated as a white powder; mp 84–85 °C.

^1^H NMR (400 MHz, CDCl_3_): 1.84–2.00 (m, 4H, CH_2_CHSe, CH_2_CHS), 2.14–2.22 (m, 2H, CH_2_CHS), 2.66–2.73 (m, 2H, CH_2_CHSe), 2.91–2.96 (m, 2H, CHS), 3.52–3.58 (m, 2H, CHSe), 3.67–3.74 (m, 4H, CH_2_S), 6.96–7.00 (m, 4H, CH_Ar_), 7.4–7.27 (m, 4H, CH_Ar_). ^13^C NMR (100 MHz, CDCl_3_): 29.2 (CH_2_CHSe), 29.7 (CH_2_CHS), 29.7 (CHSe, ^1^*J*_Se-C_ = 53.0 Hz), 34.8 (CH_2_S), 48.5 (CHS), 115.4, 115.6 (HC_Ar_), 130.2 (HC_Ar_), 134.1 (CH_2_C_Ar_), 160.7, 163.1 (FC_Ar_, ^1^*J*_F-C_ = 245.7 Hz).

Anal. calcd for C_22_H_24_F_2_S_2_Se (469.51): C 56.28, H 5.15, F 8.09, S 13.66, Se 16.82%. Found: C 55.98, H 5.06, S 13.76, Se 17.02%.

*2,6-Bis(allylsulfanyl)-9-selenabicyclo[3.3.1]nonane* (**13**) was obtained as a colourless viscous oil (0.302 g, 96% yield) from bis-isothiouronium salt **3** (0.43 g, 0.86 mmol), allyl bromide (0.315 g, 2.6 mmol) and sodium hydroxide (80%, 0.25 g, 5 mmol) in methanol under the same conditions as compound **6.**

^1^H NMR (400 MHz, CDCl_3_): 1.79–1.97 (m, 4H, CH_2_CHSe, CH_2_CHS), 2.11–2.22 (m, 2H, CH_2_CHS), 2.65–2.74 (m, 2H, CH_2_CHSe), 2.87–2.97 (m, 2H, CHS), 3.03–3.15 (m, 4H, CH_2_S), 3.49–3.56 (m, 2H, CHSe), 4.98–5.05 (dd, 4H, CH_2_=CH_,_^3^*J*_H-H_ = 9.9 Hz, ^3^*J*_H-H_ = 16.8 Hz), 5.67–5.78 (m, 2H, CH=CH_2_). ^13^C NMR (100 MHz, CDCl_3_): 29.1 (CH_2_CHSe), 29.6 (CHSe, ^1^*J*_Se-C_ = 52.9 Hz), 29.6 (CH_2_CHS), 33.9 (CH_2_S), 48.8 (CHS), 116.7 (CH_2_=CH), 134.4 (CH=CH_2_).

Anal. calcd for C_14_H_22_S_2_Se (333.41): C 50.43, H 6.65, S 19.23, Se 23.68%. Found: C 50.03, H 6.55, S 19.48, Se 23.99%.

*2,6-Bis(2-chloro-2-propenylsulfanyl)-9-selenabicyclo[3.3.1]nonane* (**14**). A solution of 2,3-dichloro-1-propene (0.289 g, 2.6 mmol) in methanol (1 mL) was added to a solution of compound **3** (0.43 g, 0.86 mmol) in methanol (5 mL). Then, a solution of sodium hydroxide (80%, 0.25 g, 5 mmol) in methanol (4 mL) was added dropwise and the mixture was heated at 50–60 °C with stirring for 7 h. Methylene chloride (20 mL) and cold water (20 mL) were added to the reaction mixture. The mixture was transferred to a separatory funnel and the organic layer was separated. The mixture was additionally extracted with methylene chloride (2 × 10 mL), the organic phase was dried over CaCl_2_ and the solvent was removed by a rotary evaporator. The residue was subjected to column chromatography on silica gel (eluent: hexane, then hexane/chloroform 1:10). Compound **14** (0.325 g, 94% yield) was isolated as a colourless viscous oil.

^1^H NMR (400 MHz, CDCl_3_): 1.89–2.01 (m, 2H, CH_2_CHSe), 2.05–2.11 (m, 2H, CH_2_CHS), 2.24–2.33 (m, 2H, CH_2_CHSe), 2.74–2.83 (m, 2H, CH_2_CHS), 3.00–3.06 (m, 2H, CHS), 3.32–3.38 (m, 2H, CH_2_S), 3.42–3.48 (m, 2H, CH_2_S), 3.65–3.69 (m, 2H, CHSe), 5.29 (s, 2H, H_2_C=CCl), 5.38 (s, 2H, H_2_C=CCl). ^13^C NMR (100 MHz, CDCl_3_): 29.4 (CH_2_CHSe), 29.8 (CH_2_CHS), 29.9 (CHSe), 39.2 (CH_2_S), 48.9 (CHS), 114.5 (H_2_C=CCl), 139.1 (ClC=CH_2_). Anal. calcd for C_14_H_20_S_2_Cl_2_Se (402.30): C 41.80, H 5.01, S 15.94, Cl 17.63, Se 19.63%. Found: C 41.66, H 4.98, S 16.07, Cl 17.51, Se 19.92%.

*2,6-Bis(2-methyl-2-propenylsulfanyl)-9-selenabicyclo[3.3.1]nonane* (**15**) was obtained from bis-isothiouronium salt **3** (0.43 g, 0.86 mmol), 3-chloro-2-methyl-1-propene, (0.313 g, 2.5 mmol) and sodium hydroxide (80%, 0.25 g, 5 mmol) in methanol under the same conditions as compound **14**. The product was purified by column chromatography on silica gel (eluent: hexane, then hexane/chloroform 1:10). Compound **15** (0.283 g, 91% yield) was isolated as a white powder; mp 52–53 °C.

^1^H NMR (400 MHz, CDCl_3_): 1.80 (s, 6H, CH_3_), 1.86–2.02 (m, 4H, CH_2_CHS, CH_2_CHSe), 2.17–2.29 (m, 2H, CH_2_CHS), 2.71–2.78 (m, 2H, CH_2_CHSe), 2.96–3.00 (m, 2H, CHS), 3.02–3.11 (m, 2H, CH_2_S), 3.14–3.21 (m, 2H, CH_2_S), 3.50–3.54 (m, 2H, CHSe), 4.79–4.83 (m, 4H, CH_2_=C). ^13^C NMR (100 MHz, CDCl_3_): 20.8 (CH_3_), 29.4 (CH_2_CHSe), 29.8 (CHSe, ^1^*J*_Se-C_ = 52.8 Hz), 29.8 (CH_2_CHS), 38.7 (CH_2_S), 48.0 (CHS), 113.4 (H_2_C=C), 141.6 (C=CH_2_).

Anal. calcd for C_16_H_26_S_2_Se (361.47): C 53.16, H 7.25, S 17.74, Se 21.84%. Found: C 52.98, H 7.22, S 17.89, Se 22.04%.

*2,6-Bis[(E)-3-phenyl-2-propenyl)sulfanyl]-9-selenabicyclo[3.3.1]nonane* (**16**) was obtained from bis-isothiouronium salt **3** (0.43 g, 0.86 mmol), (*E*)-3-chloro-1-propenylbenzene (0.365 g, 2.4 mmol) and sodium hydroxide (80%, 0.25 g, 5 mmol) in methanol under the same conditions as compound **14**. The product was purified by column chromatography on silica gel (eluent: hexane, then hexane/chloroform 1:10). Compound **16** (0.376 g, 90% yield) was isolated as a colourless viscous oil.

^1^H NMR (400 MHz, CDCl_3_): 1.99–2.19 (m, 4H, CH_2_CHS, CH_2_CHSe), 2.32–2.41 (m, 2H, CH_2_CHS), 2.88–2.95 (m, 2H, CH_2_CHSe), 3.10–3.16 (m, 2H, CHS), 3.34–3.44 (CH_2_S), 3.77–3.81 (m, 2H, CHSe), 6.22–6.34 (m, 2H, CH=CHC_Ar_), 6.52 (d, 2H, PhCH=CH, ^3^*J*_H-H_ = 16.0 Hz), 7.31–7.35 (m, 2H, CH_Ar_), 7.38–7.43 (m, 4H, CH_Ar_), 7.45–7.48 (m, 4H, CH_Ar_). ^13^C NMR (100 MHz, CDCl_3_): 29.2 (CH_2_CHSe), 29.7 (CHSe, ^1^*J*_Se-C_ = 52.8 Hz), 29.7 (CH_2_CHS), 33.4 (CH_2_S), 47.7 (CHS), 125.9 (C_Ar_), 126.2 (CH_Ar_), 127.4 (C_Vin_), 128.4 (CH_Ar_), 131.8 (C_Vin_), 136.5 (C_Ar_).

Anal. calcd for C_26_H_30_S_2_Se (485.61): C 64.31, H 6.23, S 13.21, Se 16.26%. Found: C 64.03, H 6.14, S 13.34, Se 16.43%.

*2,6-Bis(3-methoxy-3-oxo-1-propenylsulfanyl)-9-selenabicyclo[3.3.1]nonane* (**17**) (a ratio of Z/E isomers ~17:1). A solution of methyl propiolate (0.2 g, 2.28 mmol) in methanol (1 mL) was added to a solution of compound **3** (0.35 g, 0.7 mmol) in methanol (5 mL). Then, a solution of sodium hydroxide (80%, 0.1 g, 2 mmol) in methanol (3 mL) was added dropwise and the mixture was stirred overnight (15 h). Methylene chloride (15 mL) and cold water (15 mL) were added to the reaction mixture. The mixture was transferred to a separatory funnel and the organic layer was separated. The mixture was additionally extracted with methylene chloride (2 × 10 mL), the organic phase was dried over CaCl_2_ and the solvent was removed by a rotary evaporator. The residue was subjected to column chromatography on silica gel (eluent: hexane, then hexane/chloroform 1:10). Compound **17** (0.248 g, 84% yield) was isolated as a white powder; mp 165–166 °C.

(*Z*)-isomer (*Z*-**17**). ^1^H NMR (400 MHz, CDCl_3_): 1.94–2.05 (m, 2H, CH_2_CHS), 2.08–2.15 (m, 2H, CH_2_CHSe), 2.21–2.30 (m, 2H, CH_2_CHS), 2.70–2.77 (m, 2H, CH_2_CHSe), 3.04–3.10 (m, 2H, CH_2_CHS), 3.68 (s, 6H, CH_3_), 3.80–3.87 (m, 2H, CHSe), 5.82 (d, 2H, HC=CHS, ^3^*J*_H-H_ = 10.0 Hz), 7.10 (d, 2H, HC=CHS, ^3^*J*_H-H_ = 10.0 Hz). ^13^C NMR (100 MHz, CDCl_3_): 28.7 (CH_2_CHSe), 29.4 (CH_2_CHS), 30.3 (CHSe, ^1^*J*_Se-C_ = 53.8 Hz), 51.3 (CHS), 53.3 (CH_3_), 113.1 (HC=CHS), 147.6 (HC=CHS), 166.8 (COO).

(*E*)-isomer (*E*-**17**). ^1^H NMR (400 MHz, CDCl_3_): 1.94–2.05 (m, 2H, CH_2_CHS), 2.08–2.15 (m, 2H, CH_2_CHSe), 2.21–2.30 (m, 2H, CH_2_CHS), 2.70–2.77 (m, 2H, CH_2_CHSe), 3.04–3.10 (m, 2H, CH_2_CHS), 3.64 (s, 6H, CH_3_), 3.80–3.87 (m, 2H, CHSe), 5.78 (d, 2H, HC=CHS, ^3^*J*_H-H_ = 15.4 Hz), 7.59 (d, 2H, HC=CHS, ^3^*J*_H-H_ = 15.4 Hz). ^13^C NMR (100 MHz, CDCl_3_): 28.9 (CH_2_CHSe), 29.0 (CH_2_CHS), 29.3 (CHSe), 50.38 (CHS), 53.4 (CH_3_), 115.1 (HC=CHS), 145.3 (HC=CHS), 165.5 (COO).

Anal. calcd for C_16_H_22_O_4_S_2_Se (421.43): C 45.60, H 5.26, O 15.19, S 15.22, Se 18.74%. Found: C 45.34, H 5.12, S 15.34, Se 19.01%.

*2,6-Bis(3-ethoxy-3-oxo-1-propenylsulfanyl)-9-selenabicyclo[3.3.1]nonane* (**18**) (a ratio of Z/E isomers ~11:1) was obtained from bis-isothiouronium salt **3** (0.35 g, 0.7 mmol), ethyl propiolate (0.22 g, 2.24 mmol) and sodium hydroxide (80%, 0.1 g, 2 mmol) in methanol under the same conditions as compound **17**. The product was purified by column chromatography on silica gel (eluent: hexane, then hexane/chloroform 1:10). Compound **18** (0.255 g, 81% yield) was isolated as a white powder; mp 125–127 °C.

(*Z*)-isomer (*Z*-**18**). ^1^H NMR (400 MHz, CDCl_3_): 1.01–1.11 (s, 6H, CH_3_, ^3^*J*_H-H_ = 7.1 Hz), 1.78–1.90 (m, 2H, CH_2_CHS), 1.93–2.00 (m, 2H, CH_2_CHSe), 2.06–2.17 (m, 2H, CH_2_CHS), 2.56–2.63 (m, 2H, CH_2_CHSe), 2.92–2.99 (m, 2H, CH_2_CHS), 3.56–3.64 (m, 2H, CHSe), 3.93–4.02 (m, 4H, CH_3_CH_2_), 5.66 (d, 2H, (HC=CHS), ^3^*J*_H-H_ = 10.2 Hz), 7.00 (d, 2H, (HC=CHS), ^3^*J*_H-H_ = 10.2 Hz). ^13^C NMR (100 MHz, CDCl_3_): 13.9 (CH_3_), 28.3 (CH_2_CHSe), 28.9 (CH_2_CHS), 29.8 (CHSe, ^1^*J*_Se-C_ = 54.3 Hz), 52.7 (CHS), 59.6 (CH_2_O), 112.9 (SHC=CH), 147.0 (HC=CHS), 165.9 (COO).

(*E*)-isomer (*E*-**18**). ^1^H NMR (400 MHz, CDCl_3_): 1.01–1.11 (s, 6H, CH_3_, ^3^*J*_H-H_ = 7.1 Hz) 1.78–1.90 (m, 2H, CH_2_CHS), 1.93–2.00 (m, 2H, CH_2_CHSe), 2.06–2.17 (m, 2H, CH_2_CHS), 2.47–2.53 (m, 2H, CH_2_CHSe), 2.92–2.99 (m, 2H, CH_2_CHS), 3.56–3.64 (m, 2H, CHSe), 3.93–4.02 (m, 4H, CH_3_CH_2_), 5.62 (d, 2H, HC=CHS, ^3^*J*_H-H_ = 15.3 Hz), 7.44 (d, 2H, HC=CHS, ^3^*J*_H-H_ = 15.3 Hz). ^13^C NMR (100 MHz, CDCl_3_): 13.9 (CH_3_), 28.5 (CH_2_CHSe), 28.6 (CH_2_CHS), 29.1 (CHSe), 52.6 (CHS), 59.7 (CH_2_O), 114.9(HC=CHS), 144.6 (HC=CHS), 164.5 (COO).

Anal. calcd for C_18_H_26_O_4_S_2_Se (449.49): C 48.10, H 5.83, O 14.24, S 14.27, Se 17.57%. Found: C 47.96, H 5.75, S 14.41, Se 17.72%.

## 4. Conclusions

Bis-isothiouronium salt **3** was prepared in 95% yield from thiourea and 2,6-dibromo-9-selenabicyclo[3.3.1]nonane derived from the transannular addition of selenium dibromide to *cis*,*cis*-1,5-cyclooctadiene. Bis-isothiouronium salt **3** was served as valuable starting material for generation of 9-selenabicyclo[3.3.1]nonane-2,6-dithiolate anion. The latter was involved in nucleophilic substitution reactions with alkyl, benzyl and allyl halides, affording 2,6-disulfanyl-9-selenabicyclo[3.3.1]nonane derivatives **5**–**16** in 90–99% yields.

The conditions for efficient nucleophilic addition of 9-selenabicyclo[3.3.1]nonane-2,6-dithiolate anion to a triple bond of alkyl propiolates have been found. The reaction of bis-isothiouronium salt **3** with alkyl propiolates proceeded in a regio- and stereoselective manner affording 2,6-di(vinylsulfanyl)-9-selenabicyclo[3.3.1]nonanes **17** and **18** in 81–84% yields. The obtained 2,6-disulfanyl-9-selenabicyclo[3.3.1]nonane derivatives are a new family of compounds with promising biological activity.

## Data Availability

Data is available in this article and supplementary information.

## Supplementary Materials

The following are available online, the NMR spectra of the obtained compounds.

## Sample Availability

Samples of the compounds are not available from the authors.
