# Supplementary material for: Efficient Synthesis of a New Family of 2,6-Disulfanyl-9-selenabicyclo[3.3.1]nonanes
Source: Molecules. 2021 May 11;26(10):2849. doi: 10.3390/molecules26102849 (PMC8150309; doi:10.3390/molecules26102849)
Supplement: Supplementary file 1 [file molecules-26-02849-s001.zip › molecules-1209020-SI.pdf]

Supplementary Materials

Efficient Synthesis of a New Family of 2,6-Disulfanyl-9-selenabicyclo[3.3.1]nonanes

Maxim V. Musalov, Vladimir A. Potapov * and Svetlana V. Amosova

A. E. Favorsky Irkutsk Institute of Chemistry, Siberian Division of The Russian Academy of Sciences, 1 Favorsky Str., Irkutsk 664033, Russia; musalov_maxim@irioch.irk.ru (M.V.M.); amosova@irioch.irk.ru (S.V.A.)

* Correspondence: v.a.potapov@mail.ru

Table of Contents

Experimental (General Information) 2

Examples of ^{1}H and ^{13}C-NMR Spectra 3-15

Experimental (General Information)

^{1}H (400.1 MHz) and ^{13}C (100.6 MHz) NMR spectra were recorded on a Bruker DPX-400 spectrometer (Bruker BioSpin GmbH, Rheinstetten, Germany) in $CDCl_3$ (compounds 5-18) and ***d***$_6$-DMSO (compounds 3) solutions and referred to the residual solvent peaks of $CDCl_3$ (δ = 7.27 and 77.16 ppm in ^{1}H- and ^{13}C-NMR, respectively) or ***d***$_6$-DMSO (δ = 2.50 and 39.5 ppm in ^{1}H- and ^{13}C-NMR, respectively).

Examples of ^{1}H and ^{13}C-NMR Spectra

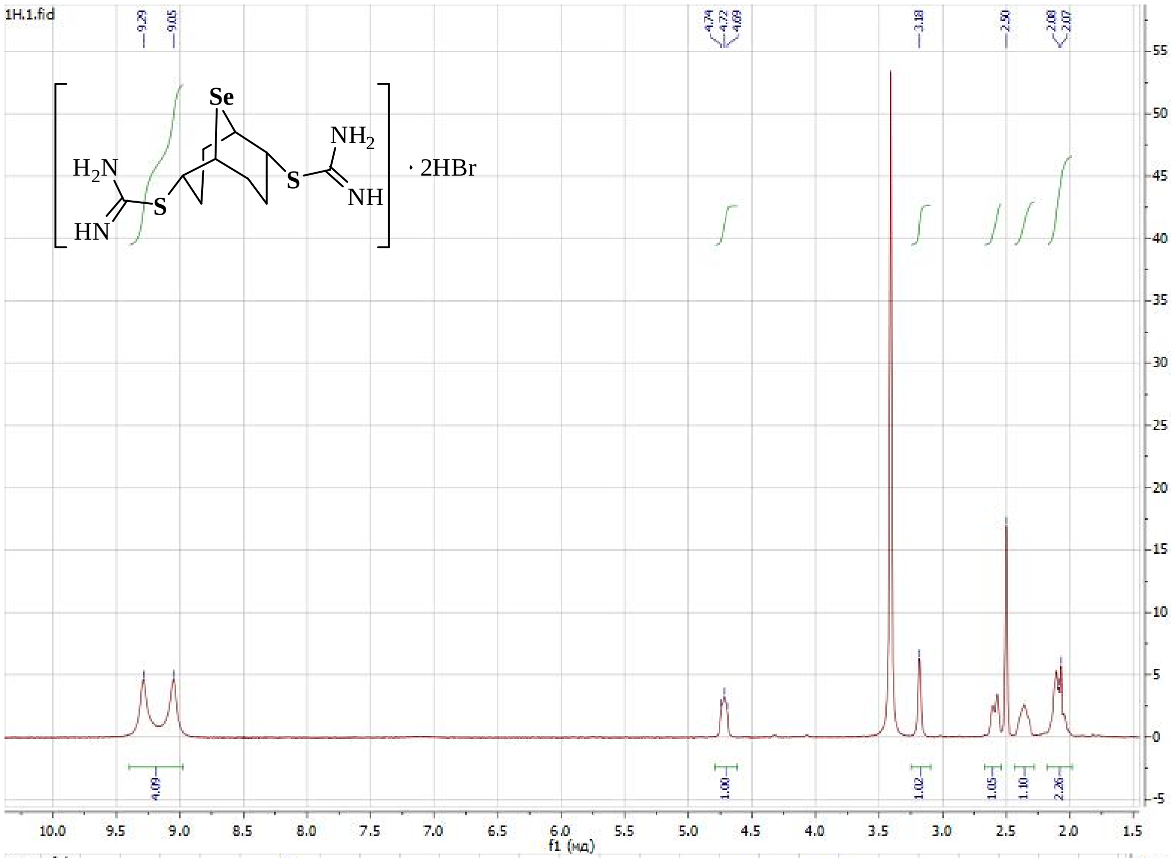

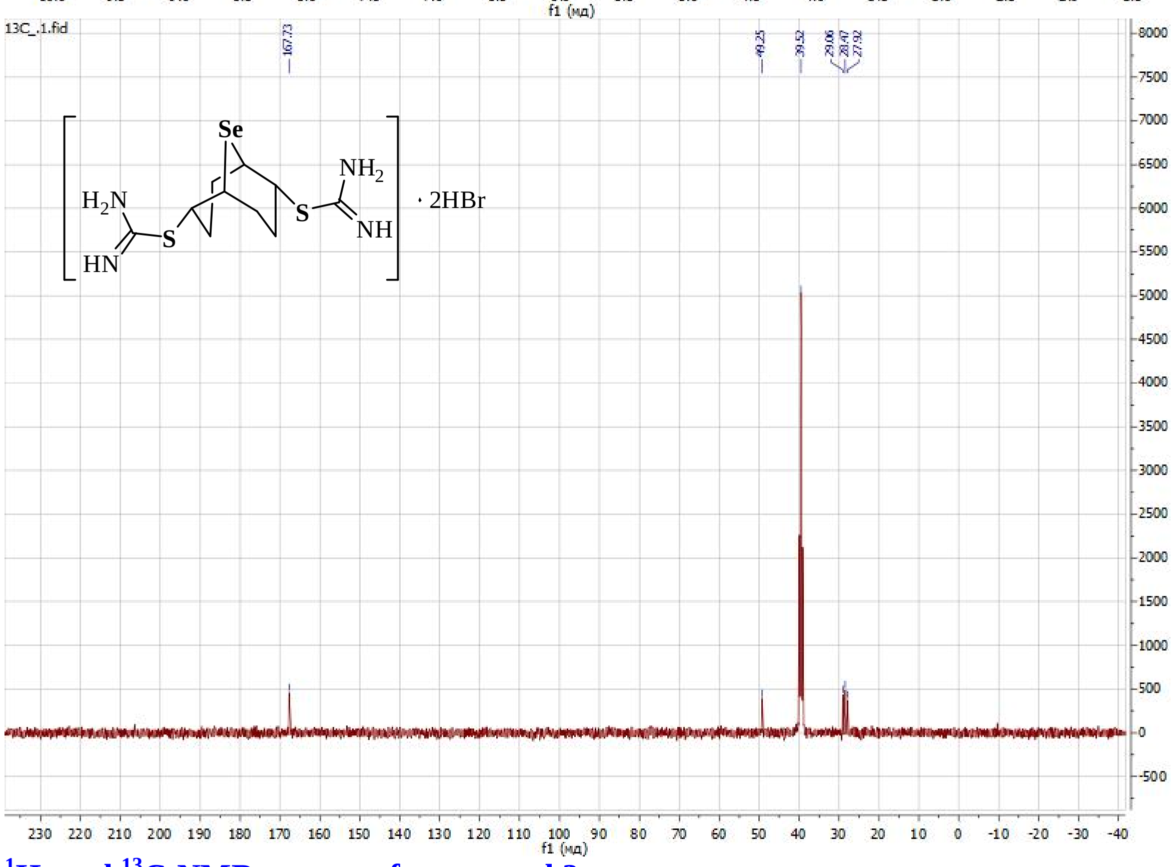

^{1}H- and ^{13}C-NMR spectra of compound 3

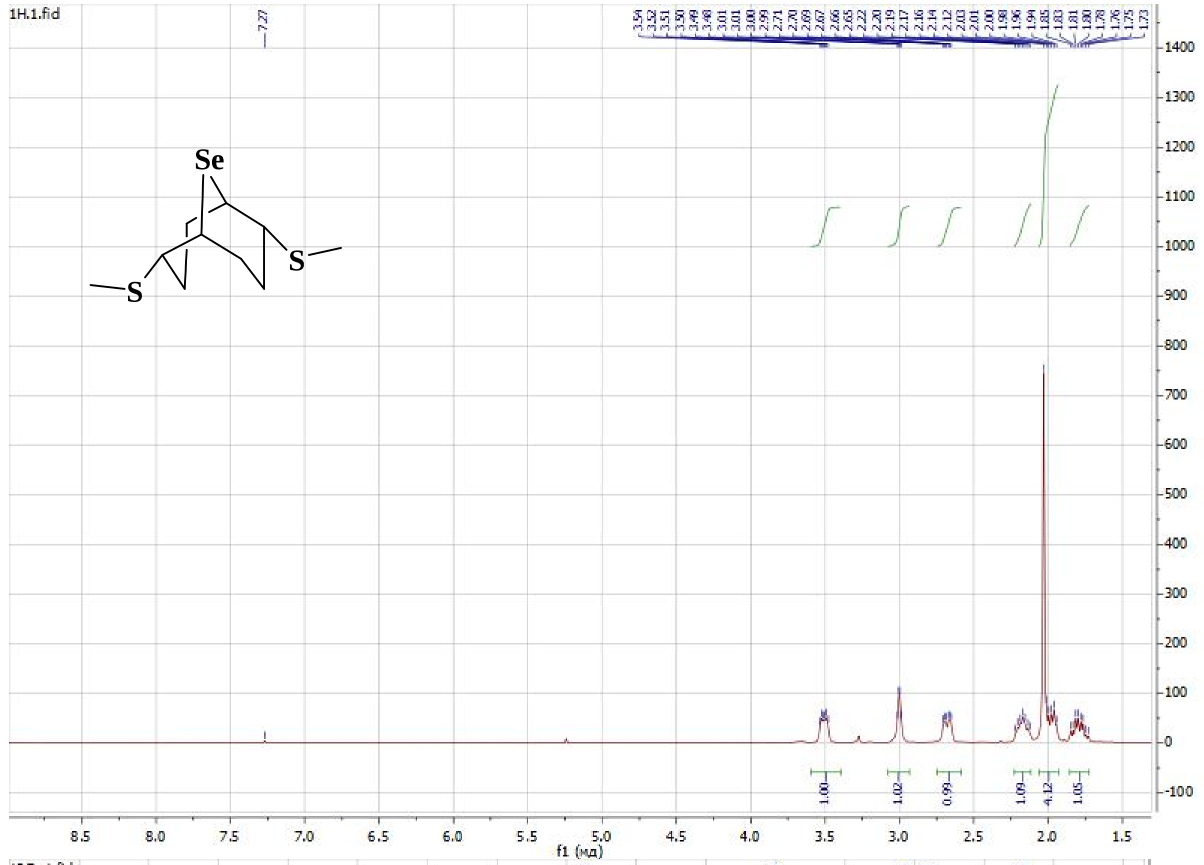

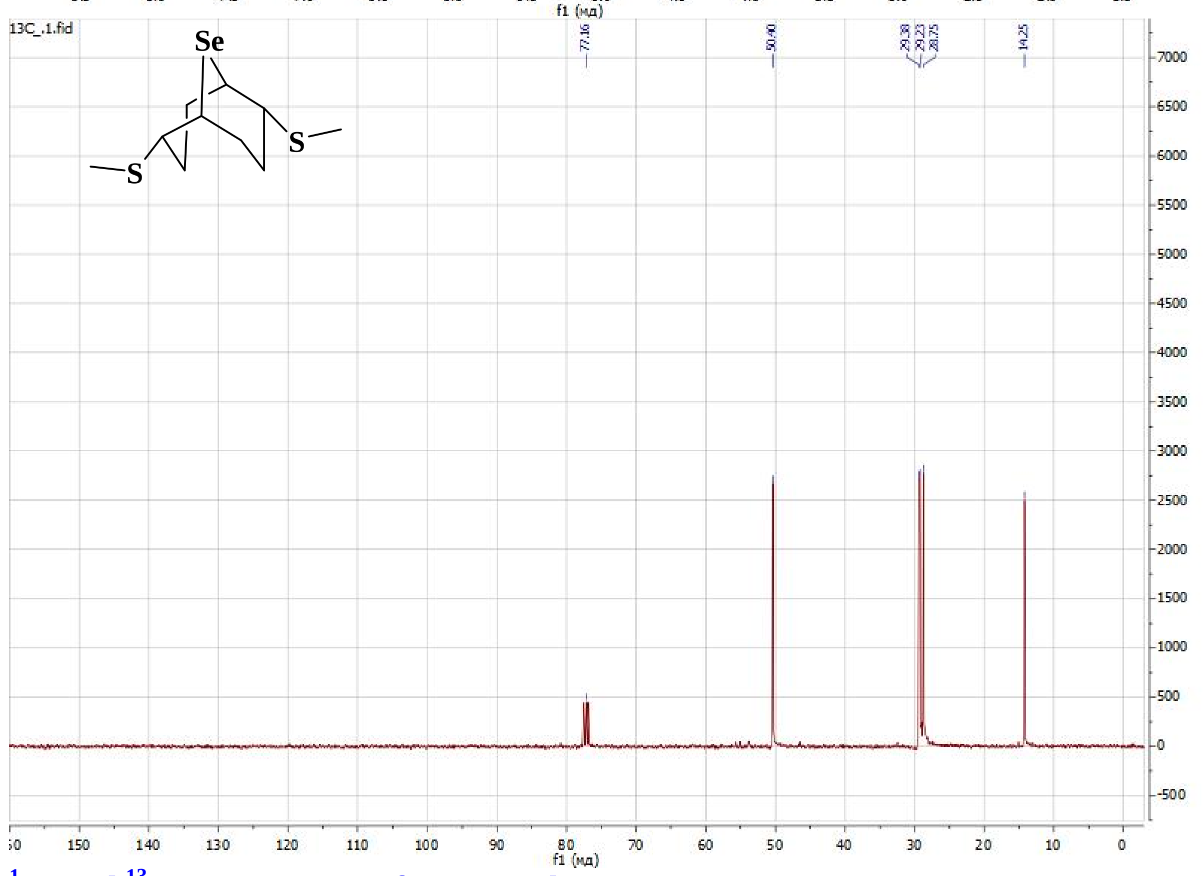

^{1}H- and ^{13}C-NMR spectra of compound 5

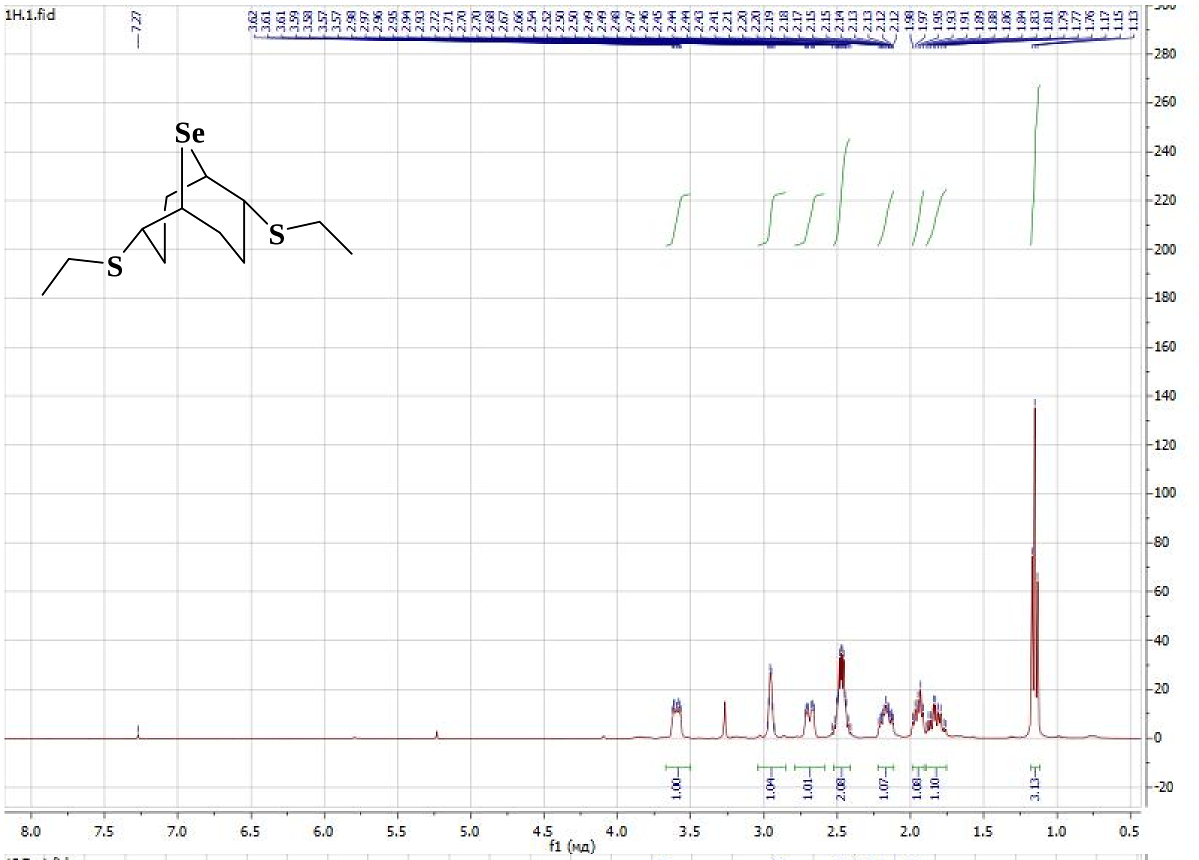

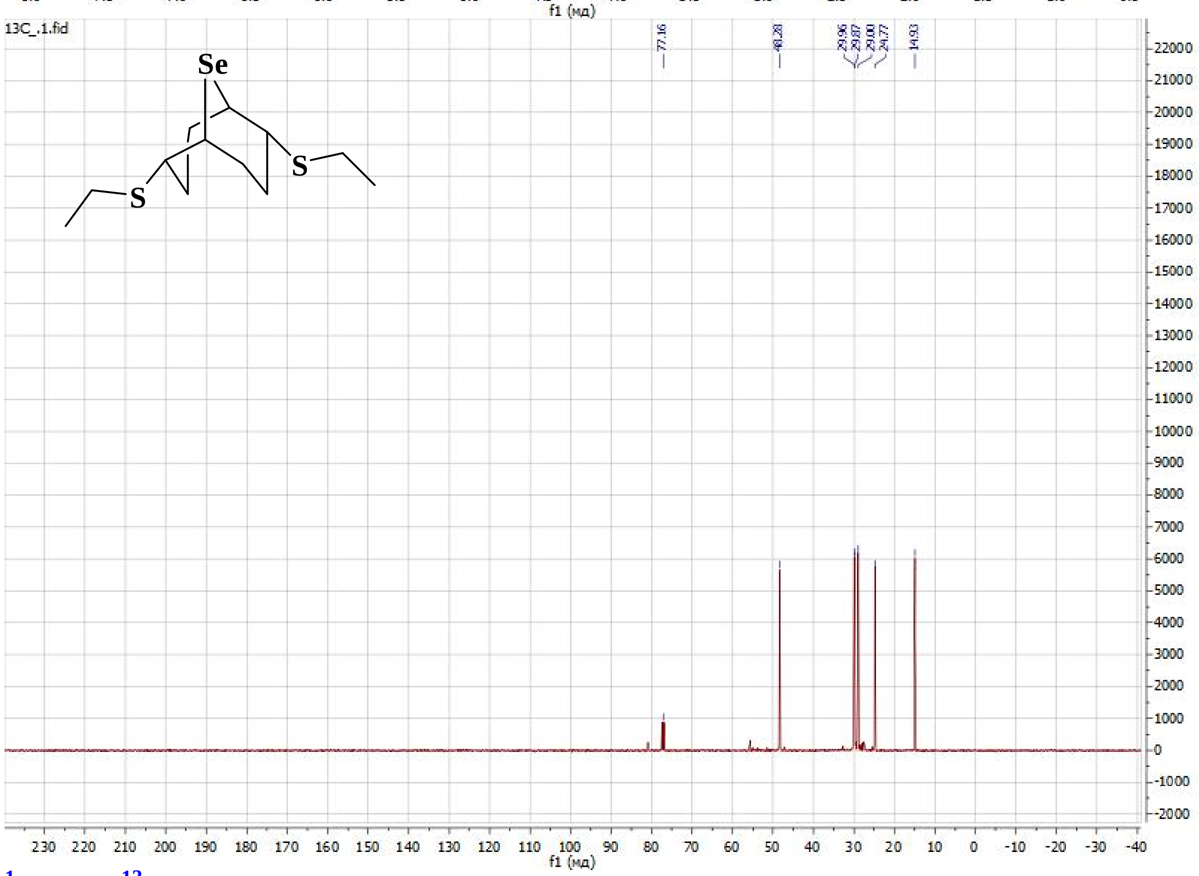

^{1}H- and ^{13}C-NMR spectra of compound 6

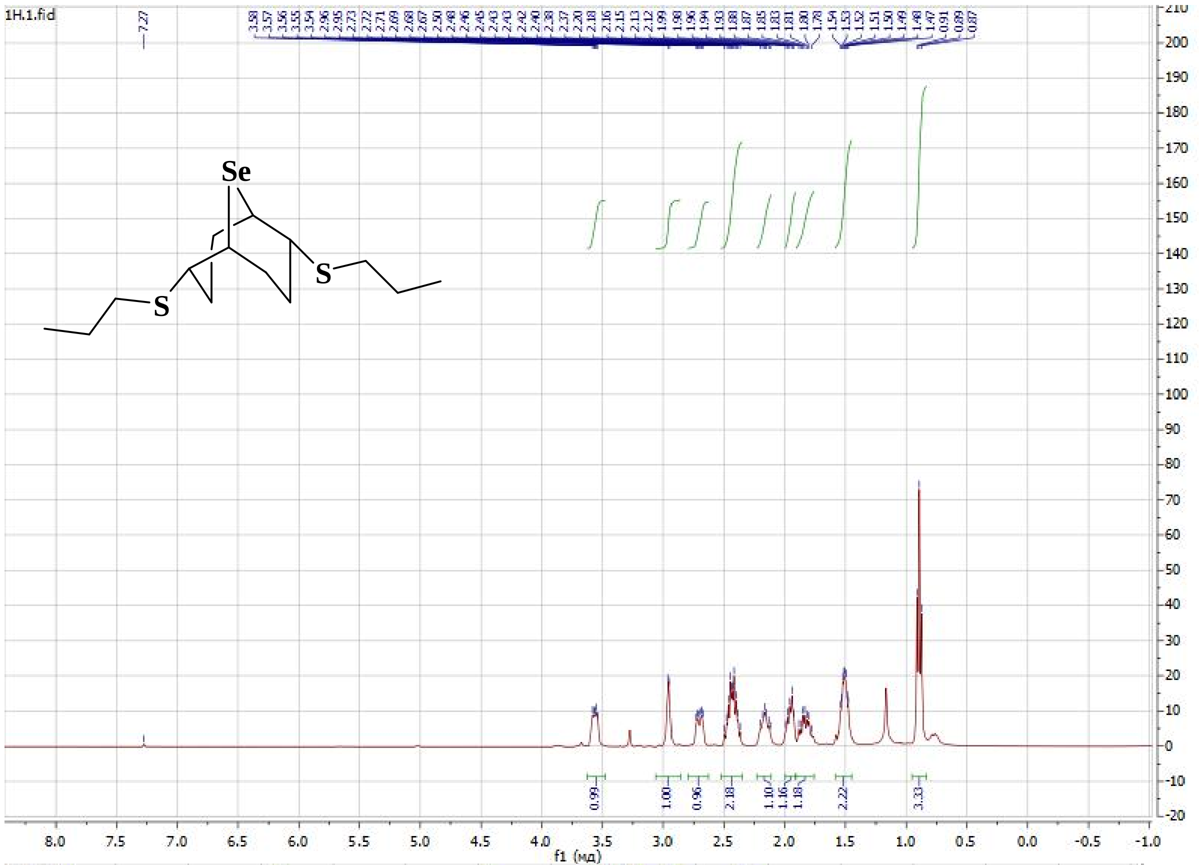

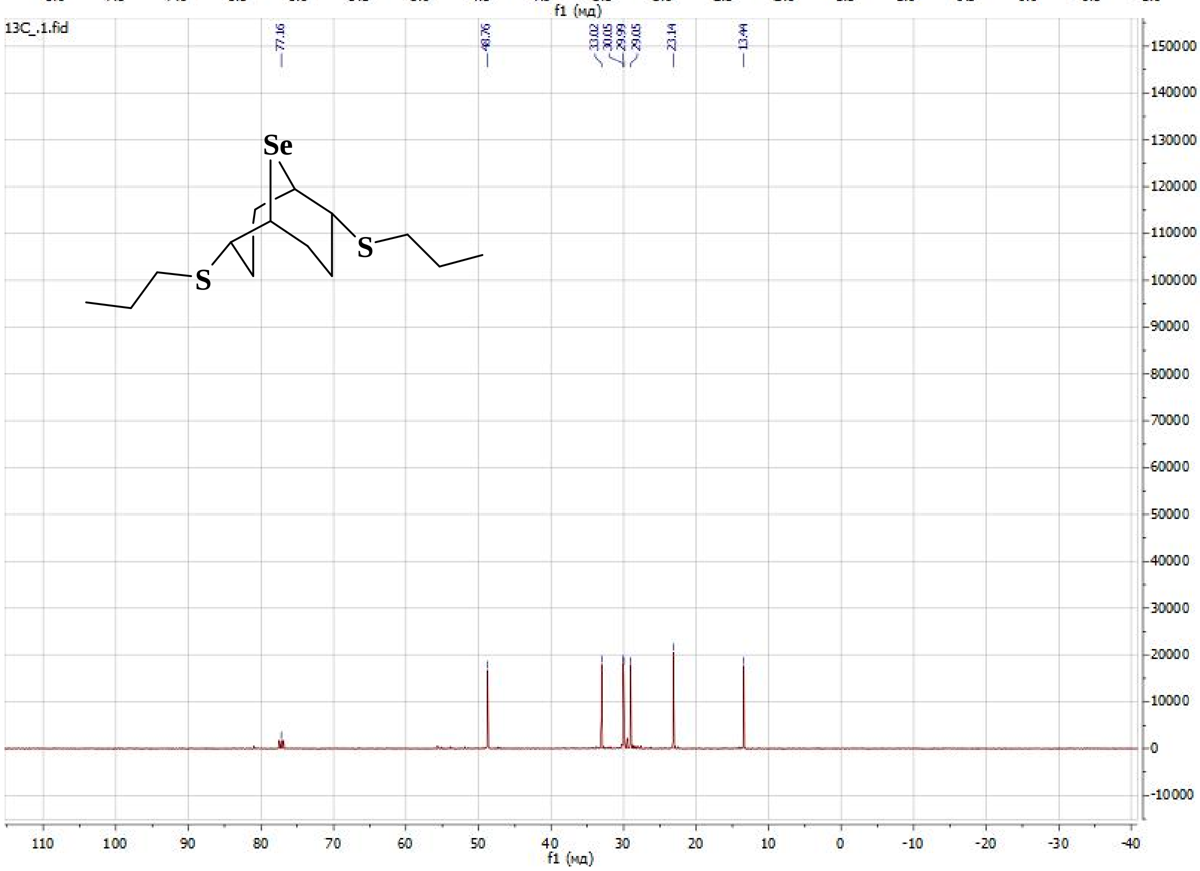

^{1}H- and ^{13}C-NMR spectra of compound 7

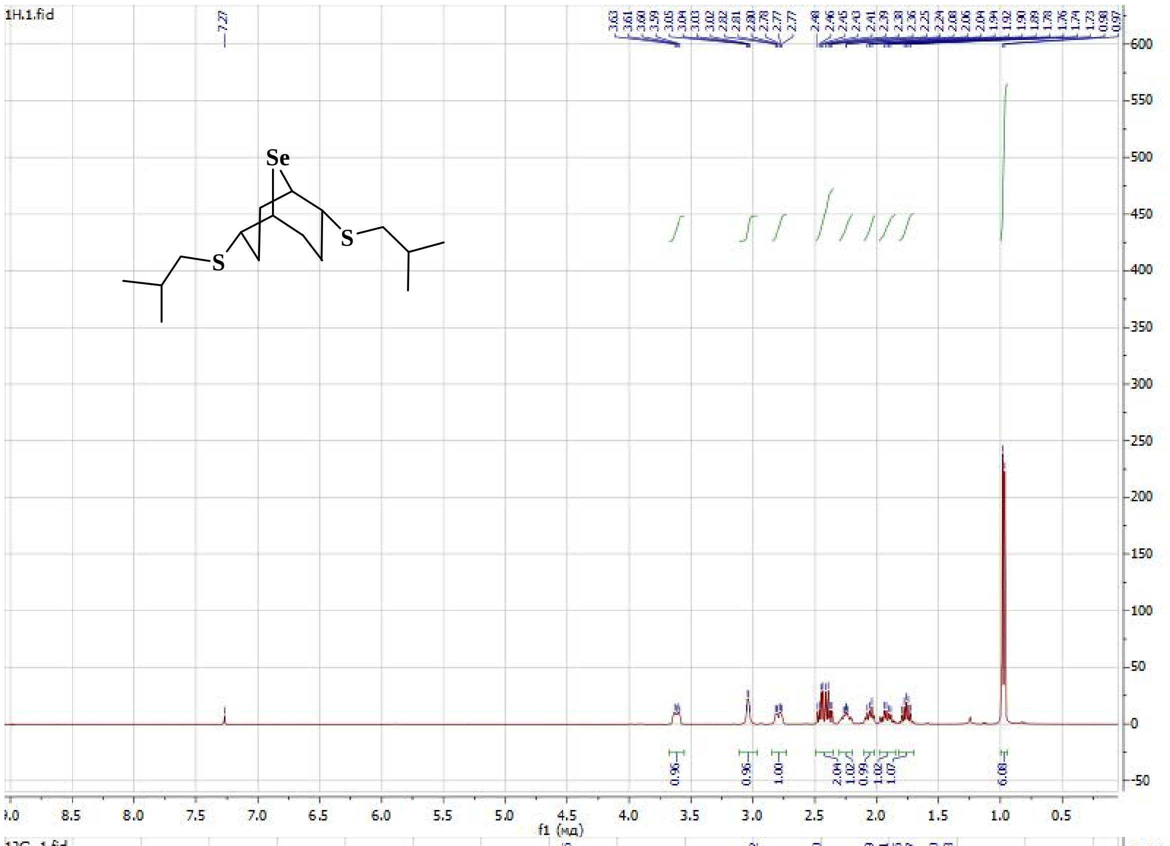

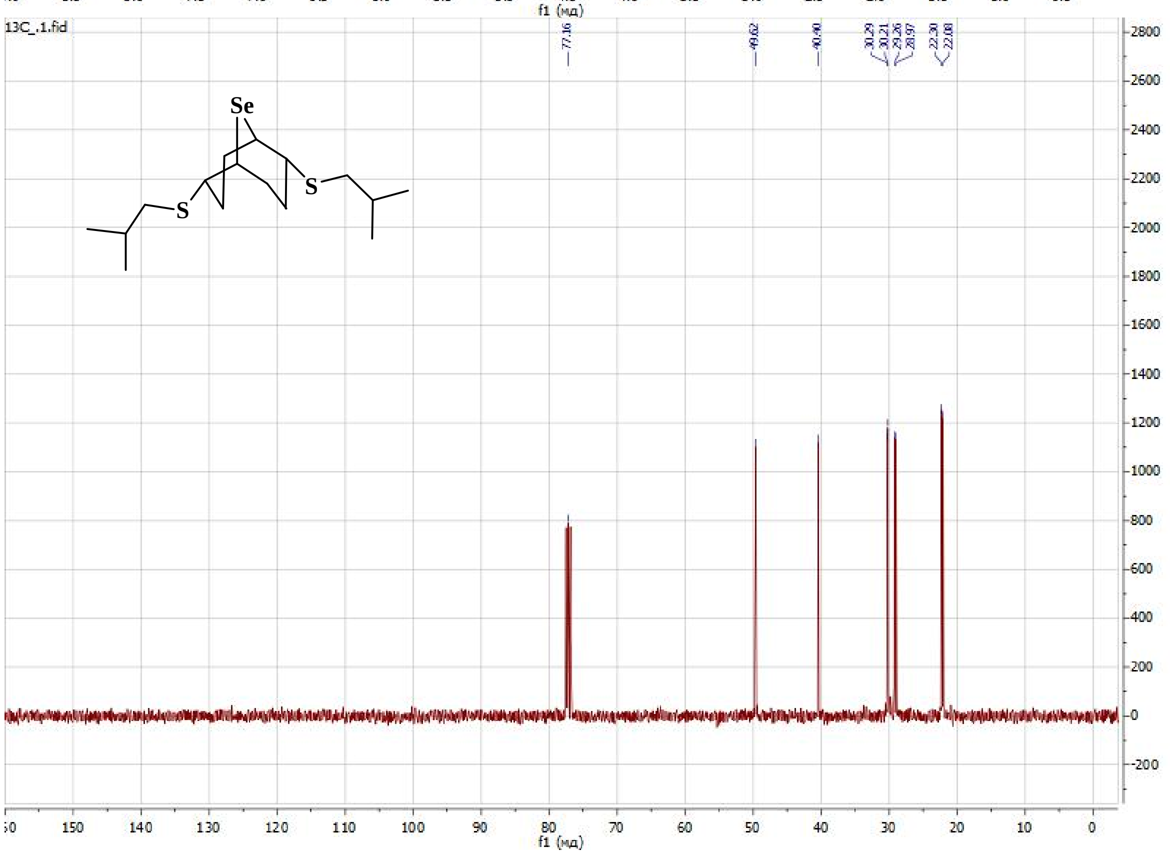

^{1}H- and ^{13}C-NMR spectra of compound 9

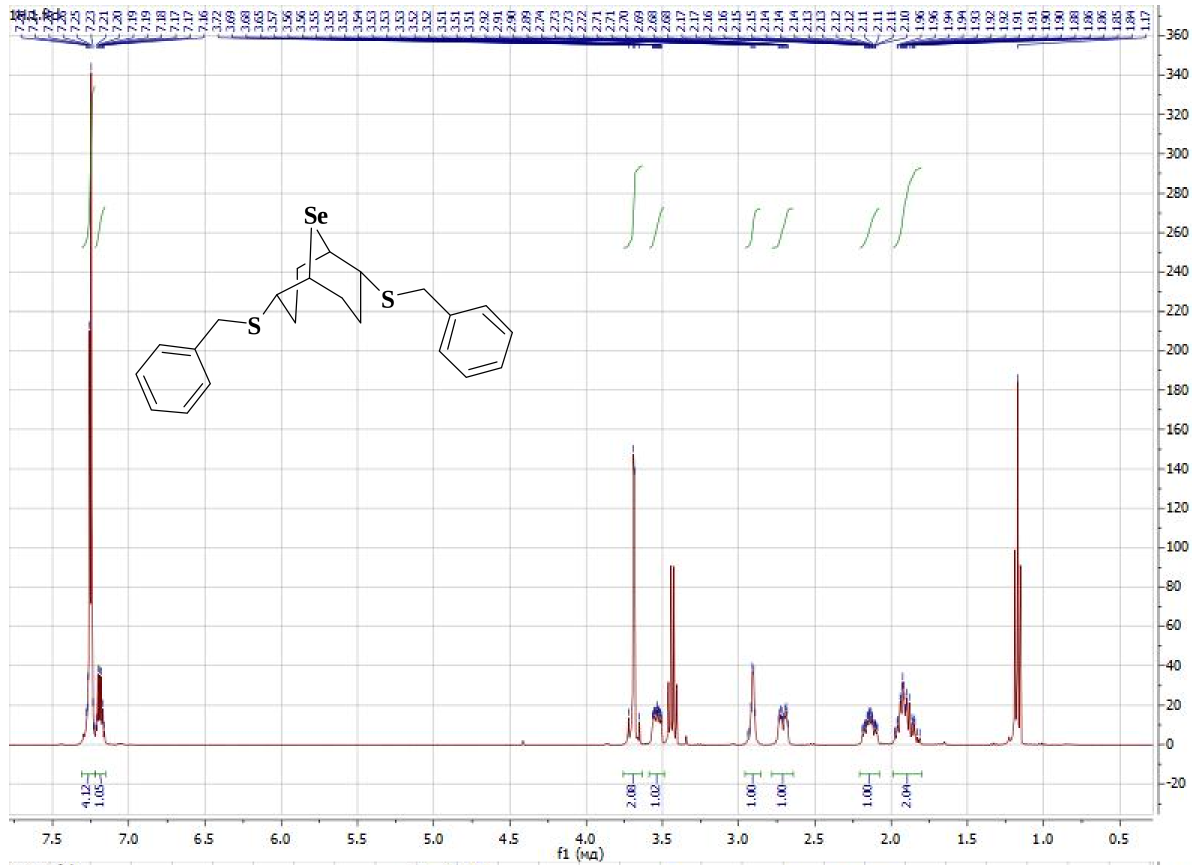

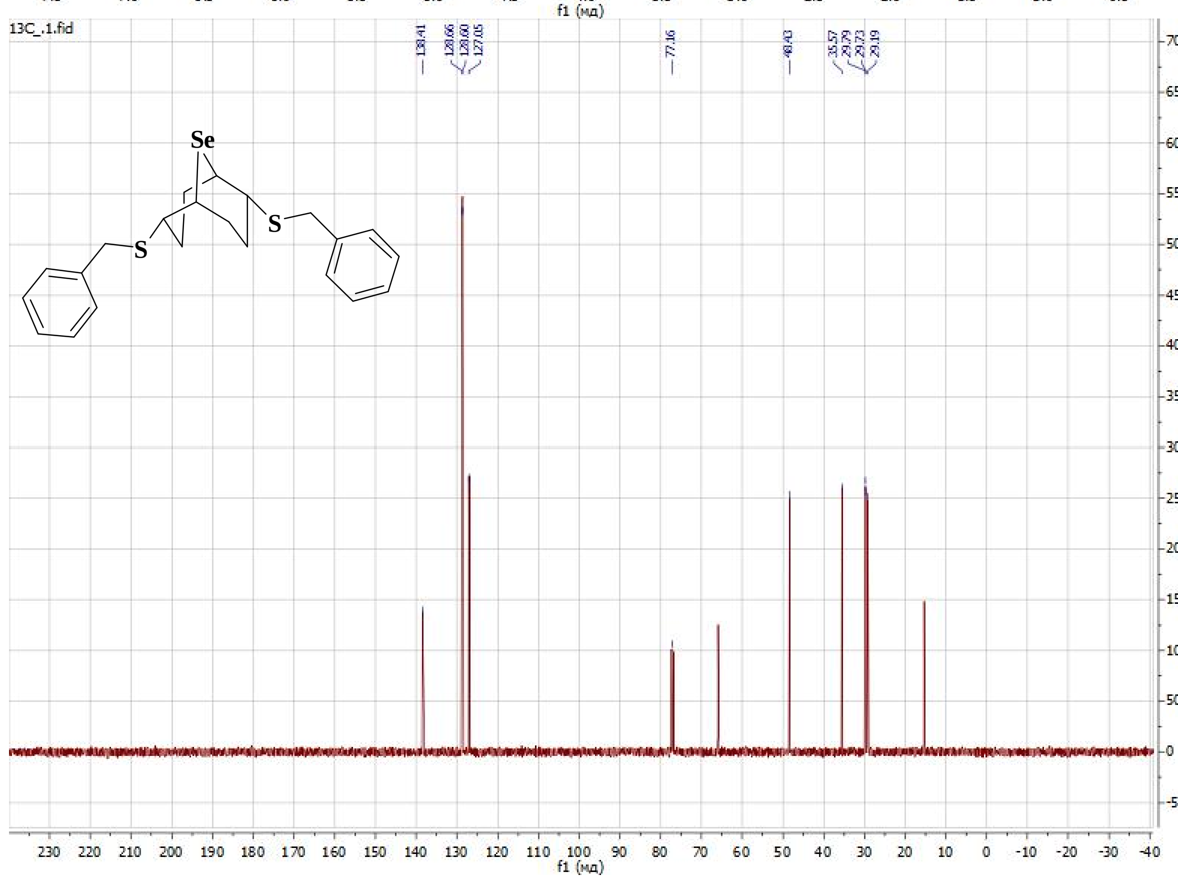

^{1}H- and ^{13}C-NMR spectra of compound 11

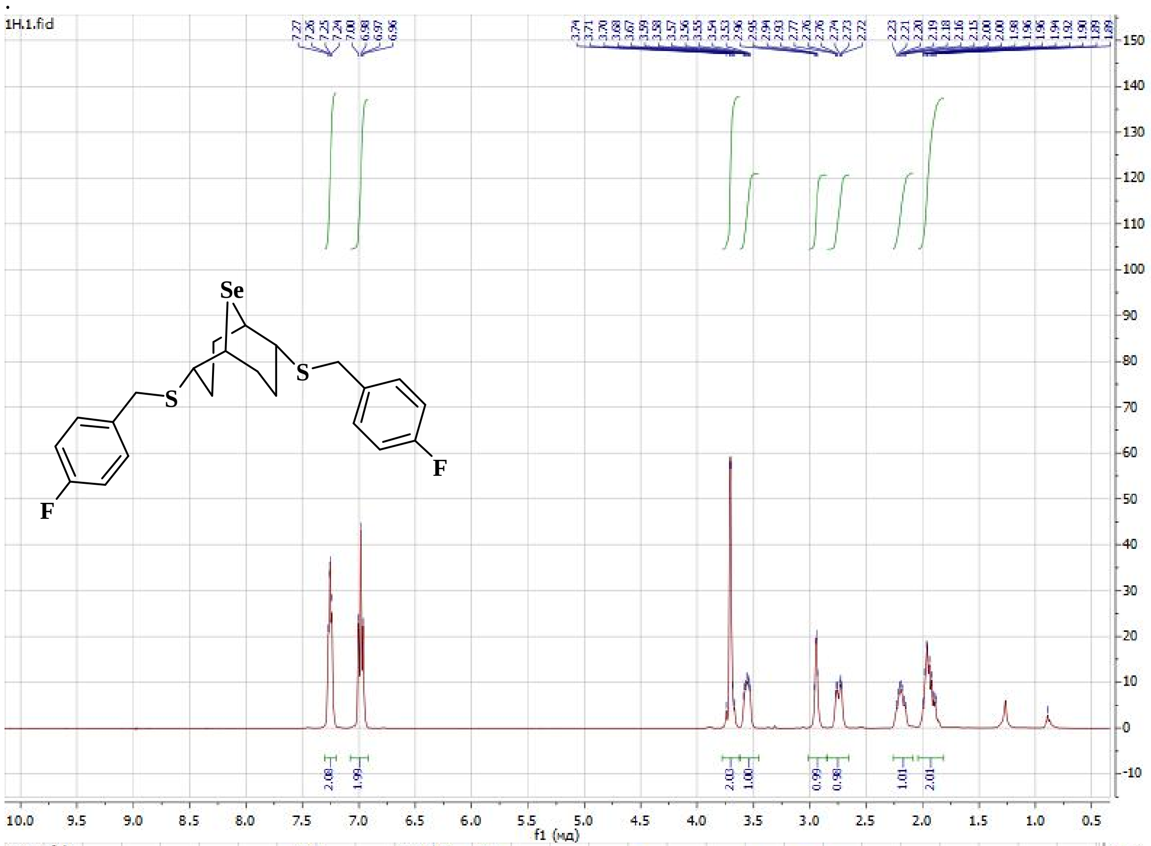

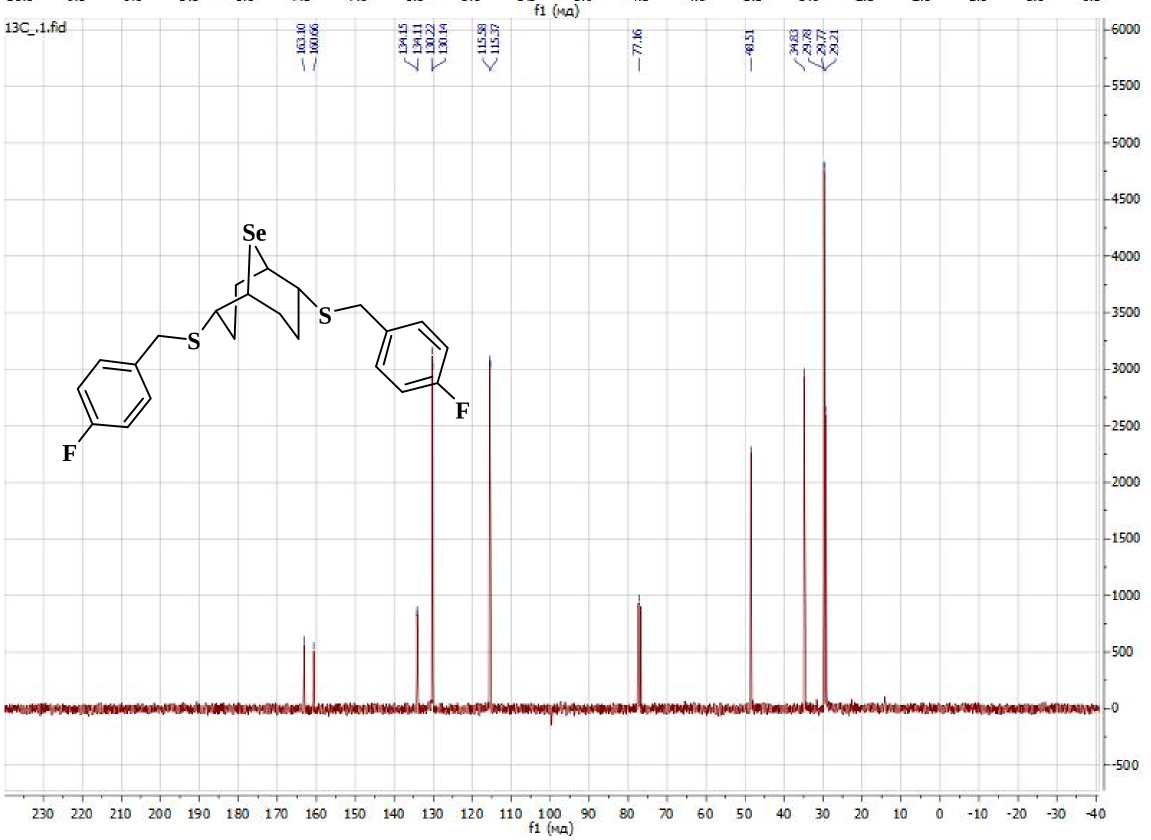

^{1}H- and ^{13}C-NMR spectra of compound 12

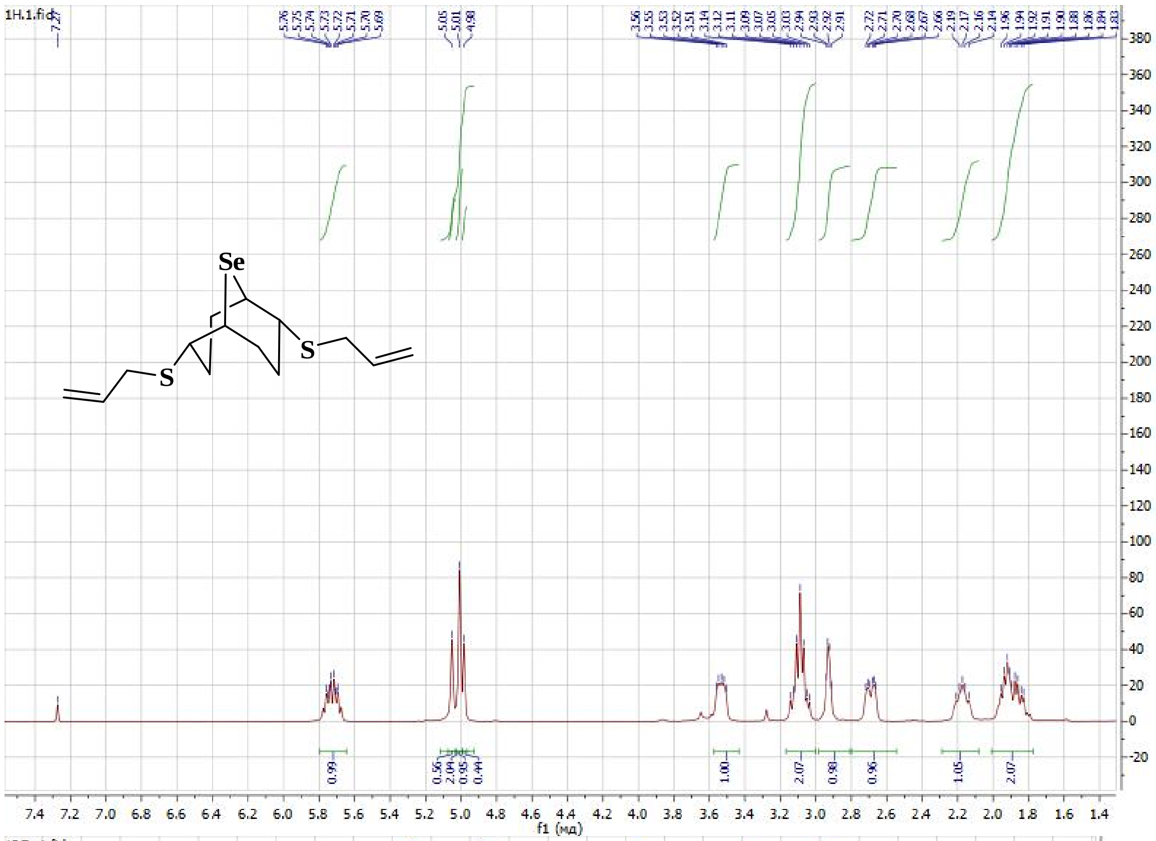

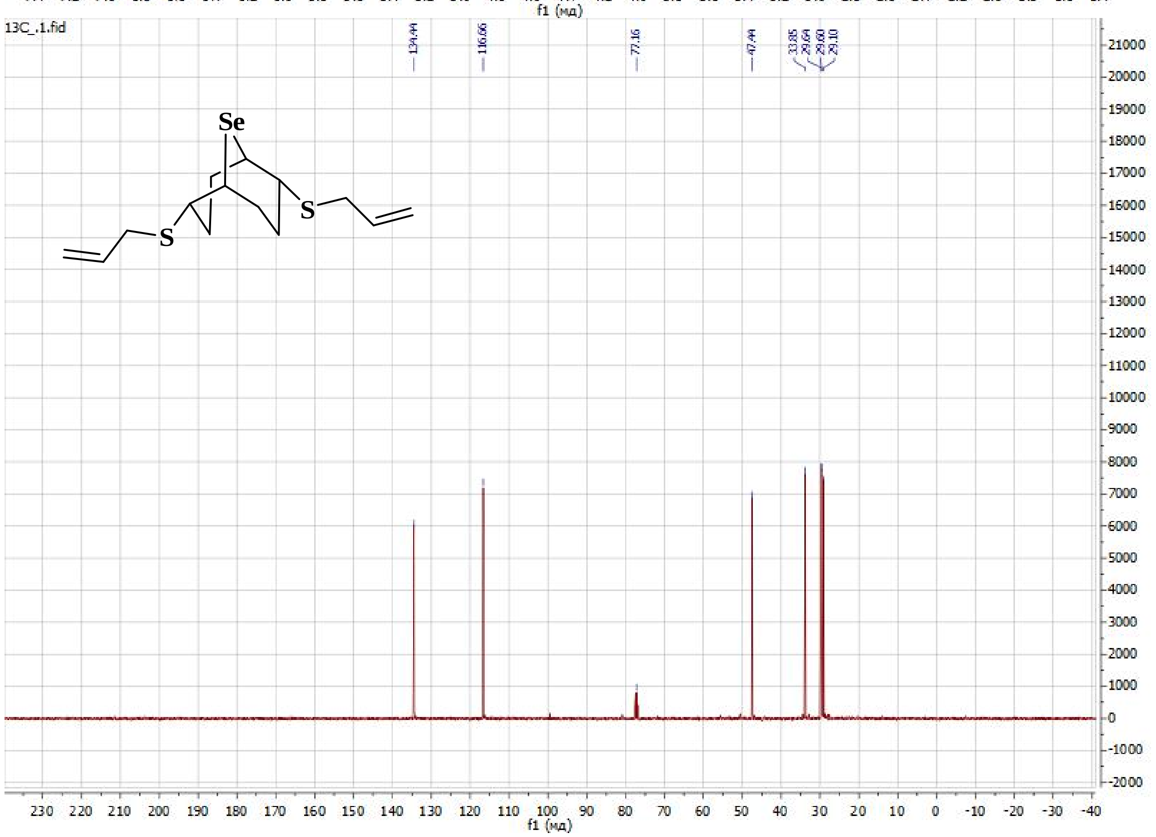

^{1}H- and ^{13}C-NMR spectra of compound 13

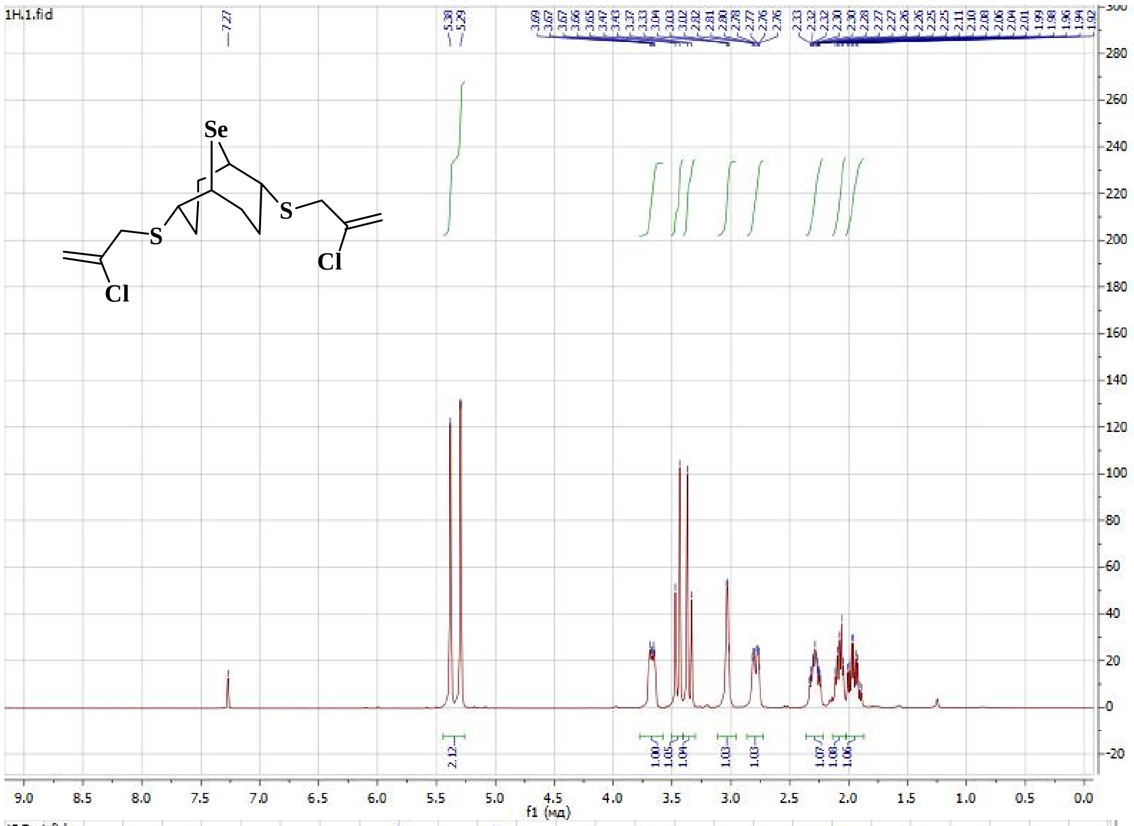

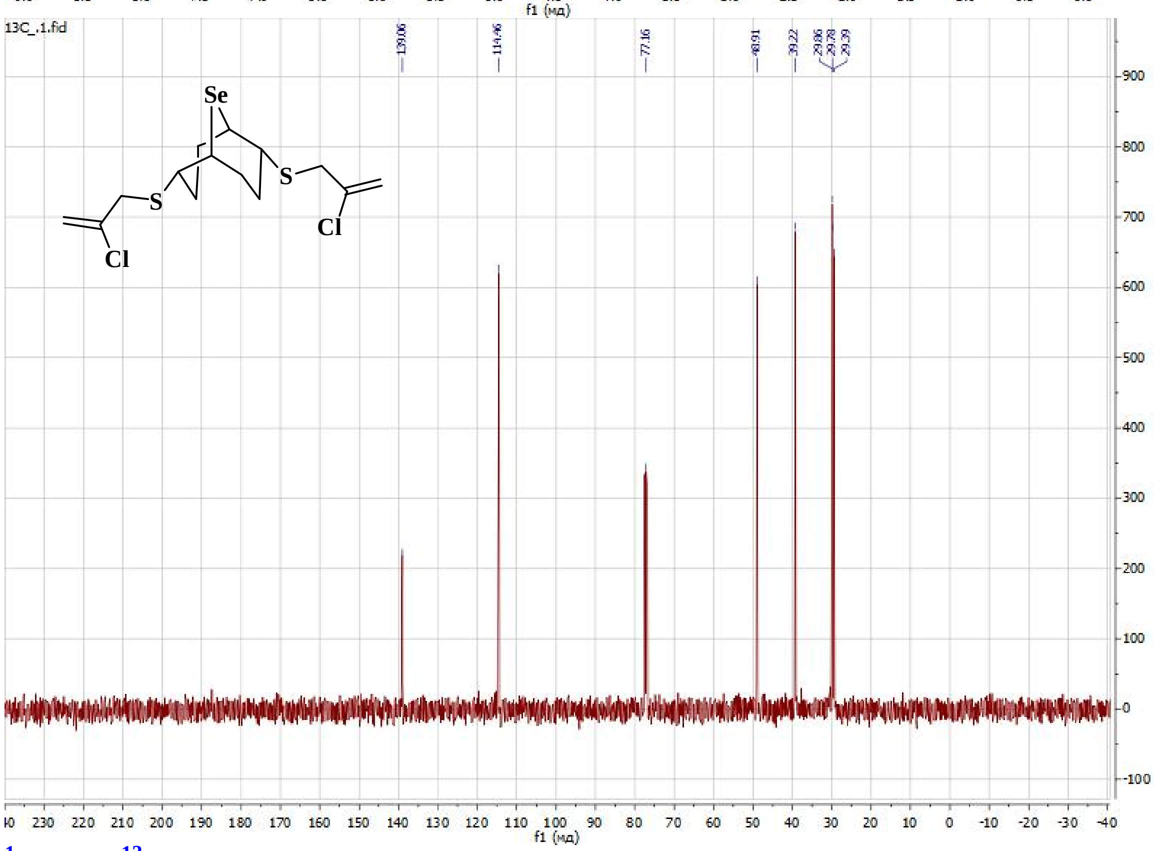

^{1}H- and ^{13}C-NMR spectra of compound 14

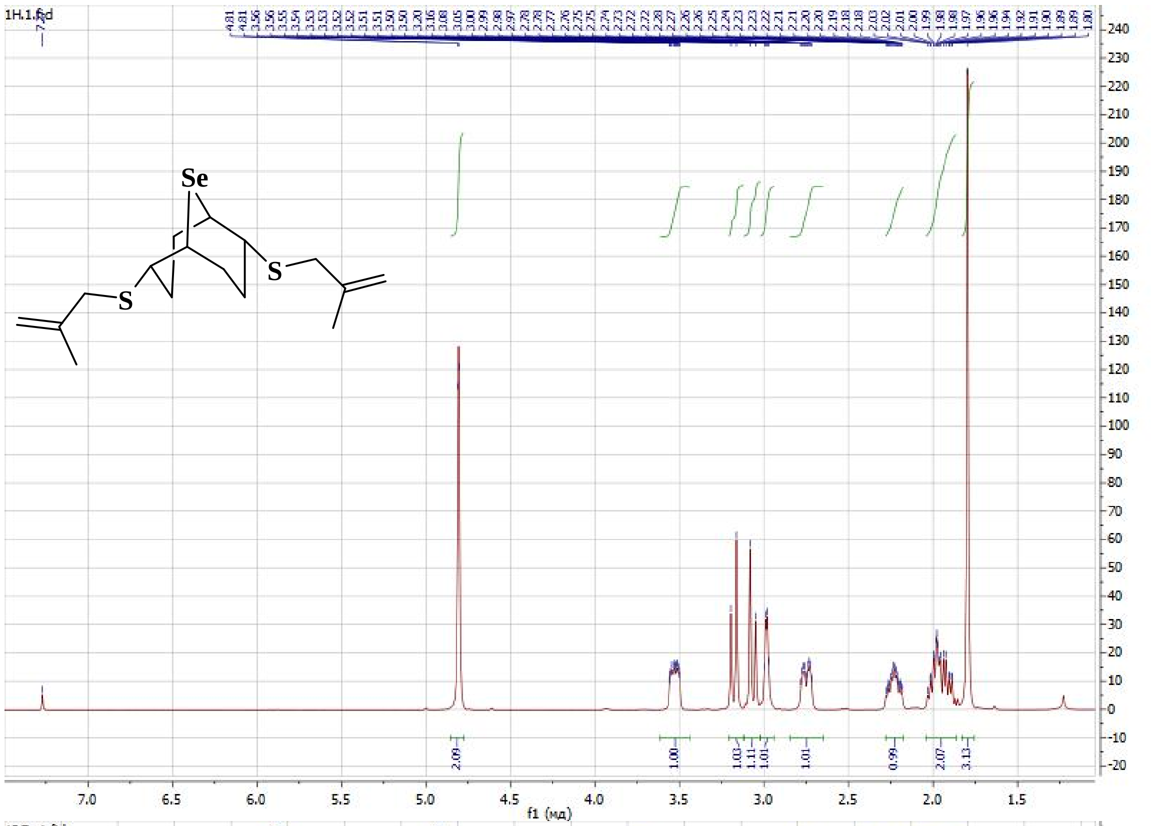

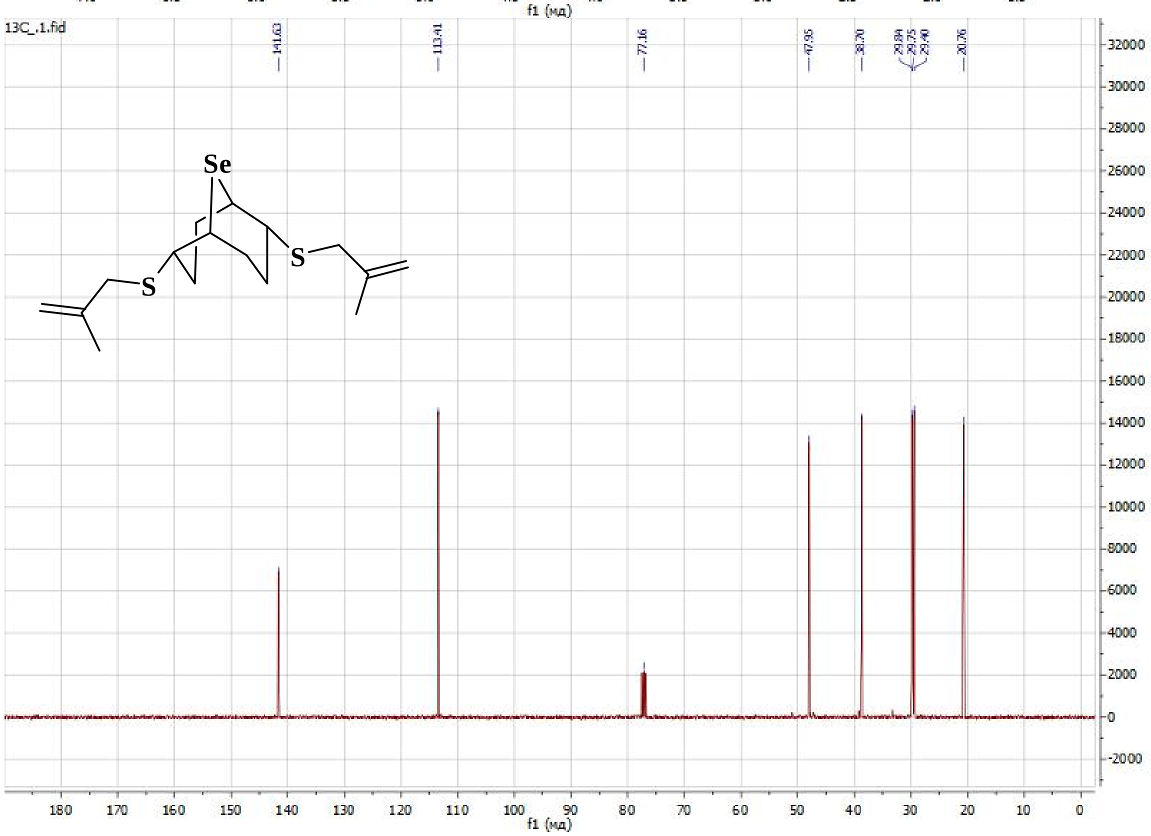

^{1}H- and ^{13}C-NMR spectra of compound 15

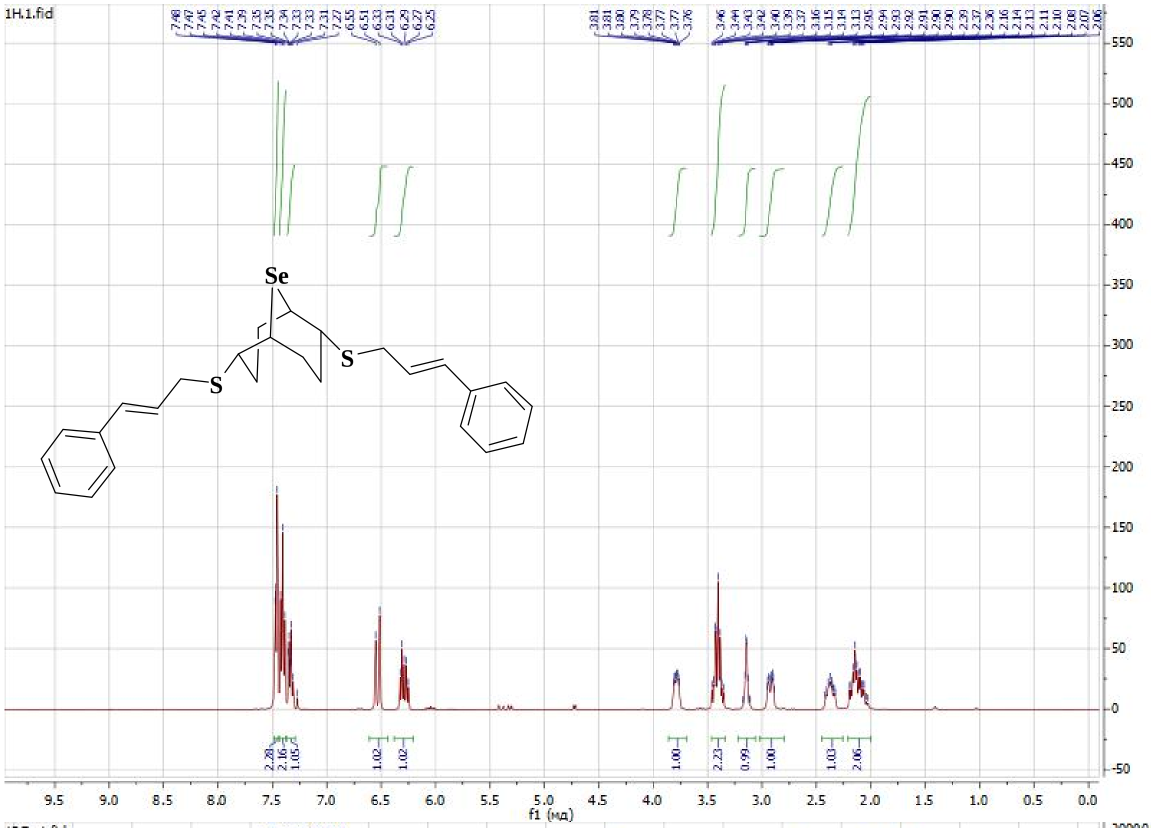

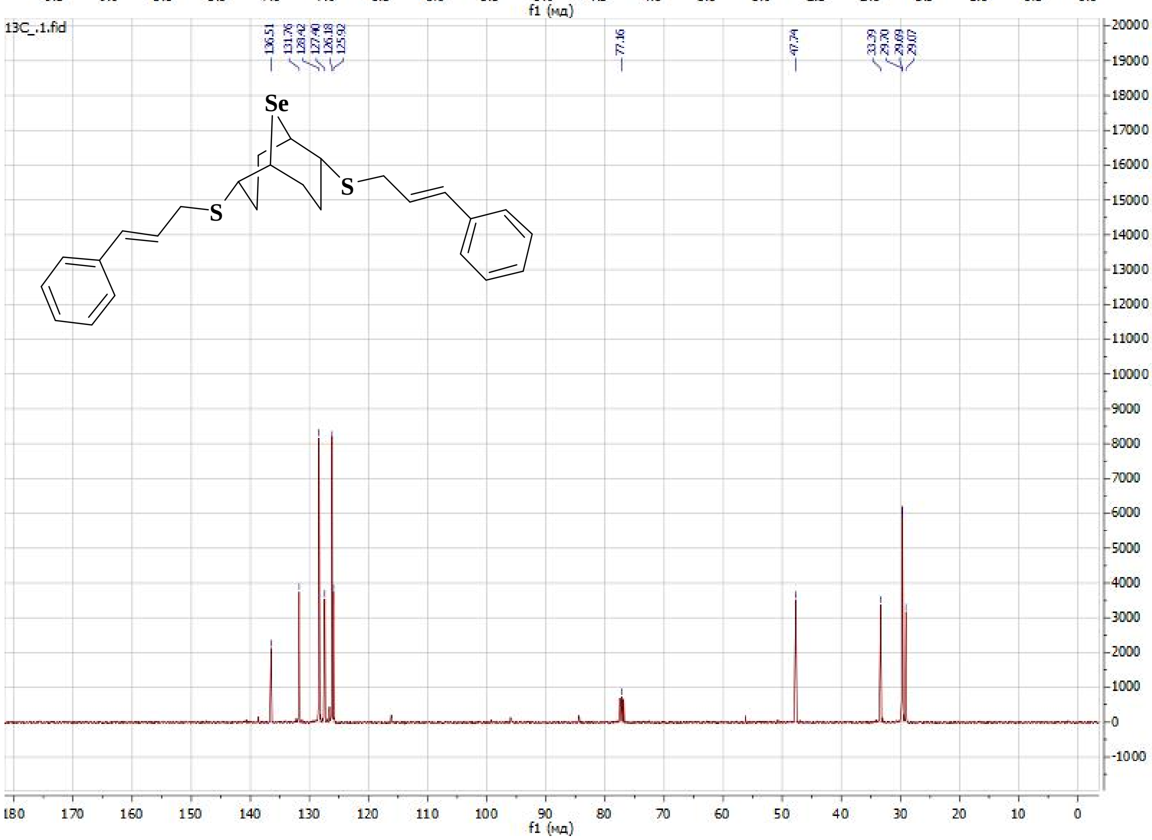

^{1}H- and ^{13}C-NMR spectra of compound 16

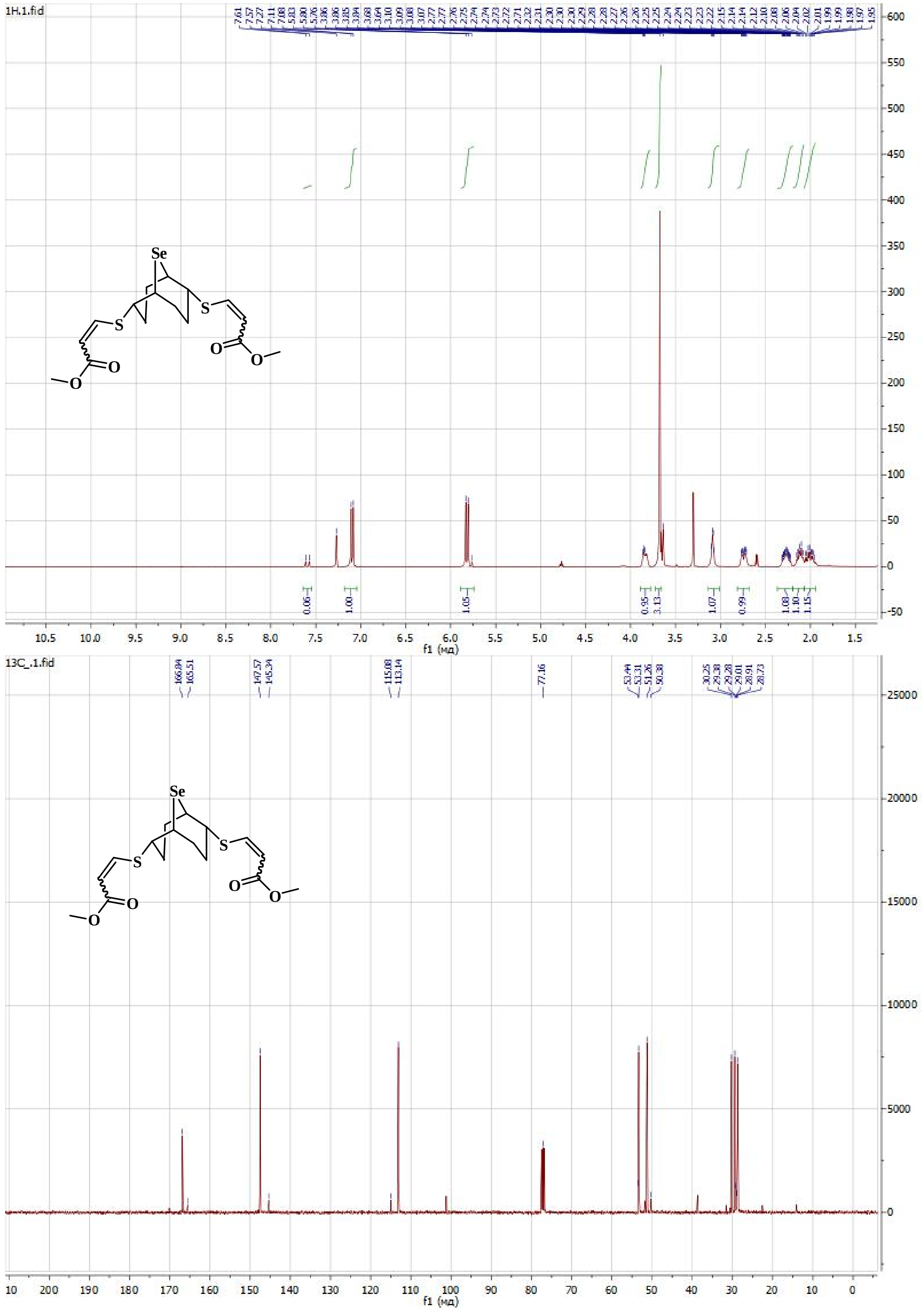

^{1}H- and ^{13}C-NMR spectra of compound 17 (a ratio of Z/E isomer ~ 17: 1)

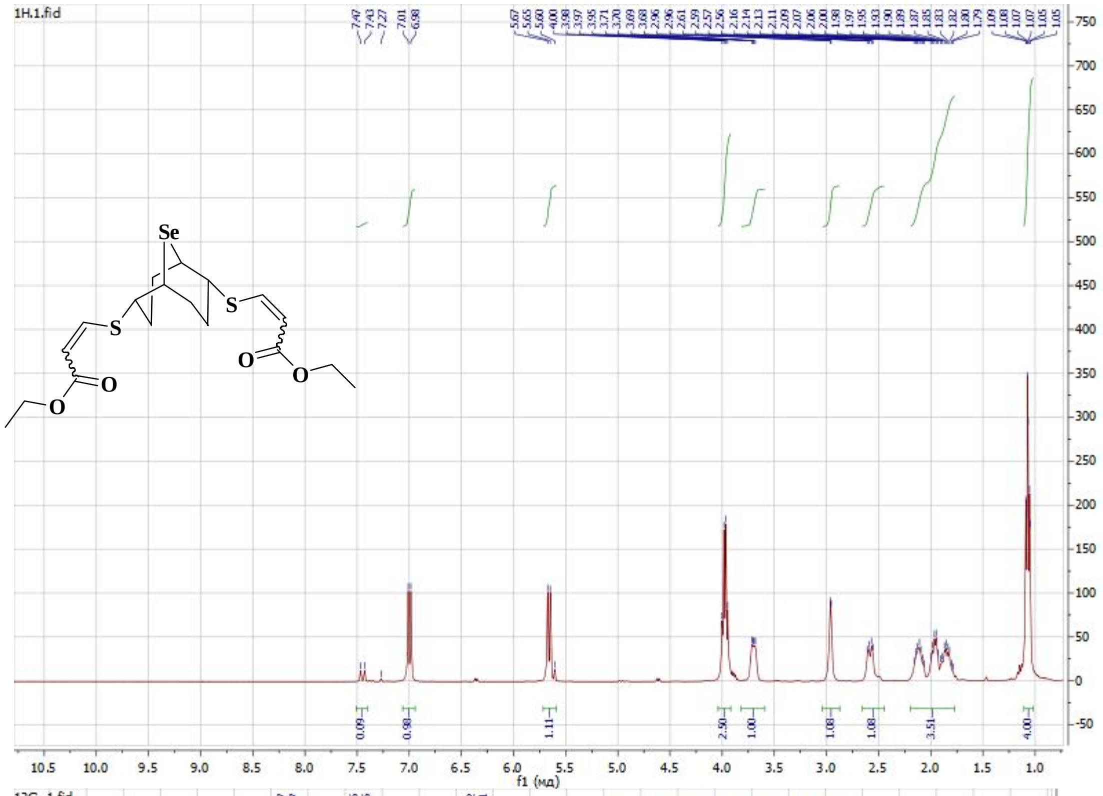

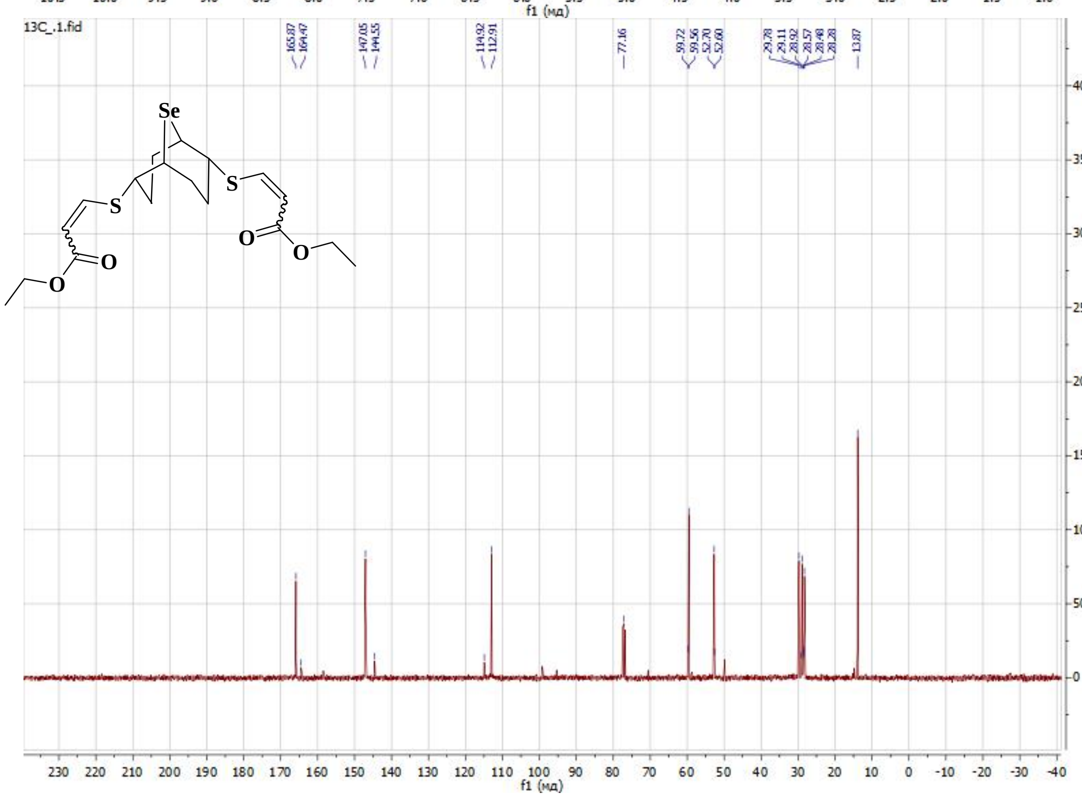

^{1}H- and ^{13}C-NMR spectra of compound 18 (a ratio of Z/E isomer ~ 11: 1)
